# Multi-omics integration and machine learning reveal gut-immune signatures in idiopathic pulmonary fibrosis: insights from bulk RNA-seq, single-cell profiles, spatial transcriptomics, and experimental validation

**DOI:** 10.3389/fimmu.2026.1730289

**Published:** 2026-03-19

**Authors:** Zhengyu Hu, Jiaqi Wang, Jialin Yu, Zheqing Hu, Jing Xue, Zhanbing Ma, Miaomiao Nian, Ruixin Qi, Tingting Zhao, Xia Cao, Hongxia Xin, Xiuyan Wang, Guilan Yang, Zhenzhen Gui, Xiaoming Liu, Juan Chen

**Affiliations:** 1 Ningxia Key Laboratory of Clinical and Pathogenic Microorganisms, Institute of Medical Science, General Hospital of Ningxia Medical University, Yinchuan, Ningxia, China; 2 Department of Respiratory and Critical Care Medicine, General Hospital of Ningxia Medical University, Yinchuan, China; 3 Department of Anatomy and Cell Biology, University of Iowa, Iowa City, IA, United States; 4 Department of Medical Genetics and Cell Biology, College of Basic Medicine, Ningxia Medical University, Yinchuan, China

**Keywords:** gut microbiota, idiopathic pulmonary fibrosis, immune cells, lung–gut axis, machine learning, multi-omics, spatial transcriptomics

## Abstract

**Background:**

Idiopathic pulmonary fibrosis (IPF) is a progressive, fatal lung disease with limited treatment options and a poor prognosis. Recent studies suggest a critical role for the gut–immune–lung axis in IPF, yet the underlying molecular mechanisms remain unclear.

**Methods:**

The current study performed in silico multi-omics integration of publicly available datasets, including bulk RNA-seq, single-cell and spatial transcriptomics, as well as peripheral blood multi-omics data to uncover key molecular signatures in IPF. Furthermore, machine learning techniques were utilized to identify core genes, whereas functional analyses and Mendelian randomization were conducted to evaluate the causal relationships among gut microbiota, immune cells, and IPF. Additionally, experimental validation using qPCR and ELISA assays was conducted *in vitro*, *in vivo*, and in patient plasma to confirm the expression patterns of key genes.

**Results:**

Across integrated public bulk, single-cell, spatial, and blood multi-omics, CXCL13, IL33, TLR4, and IGF1 were identified as core IPF genes consistently linked to immune infiltration and fibrotic remodeling. Deconvolution, scRNA-seq, and spatial mapping localized their dysregulation to fibroblasts and immune compartments (notably B-cell, macrophage, and mast-cell axes), highlighting fibroblast–immune crosstalk in fibrotic foci. A four-gene model robustly distinguished IPF from controls across cohorts. Mendelian randomization supported a gut–immune–lung axis, indicating causal effects of specific gut taxa on IPF risk via immune phenotypes. qPCR/ELISA in TGF-β1–stimulated fibroblasts, bleomycin mouse lungs, and patient plasma corroborated upregulation of IL33, CXCL13, IGF1 and downregulation of TLR4. Drug-signature reversal nominated cucurbitacin I and temsirolimus; molecular docking was performed as a preliminary in silico, computer-simulation–based assessment of potential ligand–protein interactions between these compounds and the four core targets.

**Conclusion:**

This study provides new insights into the importance of gut–immune–lung axis in IPF and identifies CXCL13, IL33, TLR4, and IGF1 as diagnostic signatures and therapeutic targets. By integrating public multi-omics resources with experimental validation, our findings offer a foundation for future diagnostic and treatment strategies aimed at modulating the gut microbiota and immune system in IPF.

## 1 Introduction

Idiopathic pulmonary fibrosis (IPF) is a chronic, progressive interstitial lung disorder of undetermined etiology that primarily impacts the elderly population (1, 2). It is distinguished by the progressive scarring of the lung parenchyma and diffuse fibrotic changes, which result in irreversible impairments in ventilation and gas exchange functions (3). Even when treated with antifibrotic therapies, such as pirfenidone or nintedanib, the median survival time after diagnosis is merely approximately 3 to 5 years (4, 5). On a global scale, the incidence of IPF is on the rise, posing an increasing burden on public health (6, 7). The current consensus posits that genetically predisposed hosts, upon exposure to repeated micro-injuries, initiate an abnormal wound-healing response. In this process, fibroblasts and myofibroblasts form fibroblastic foci within the lungs, and excessive collagen deposition leads to over-remodeling of the extracellular matrix, ultimately resulting in architectural distortion (8). Nevertheless, the initiating and sustaining signals that drive this pathological process remain to be elucidated. Recent research suggests that the “gut–lung axis” assumes a crucial function in the initiation and advancement of IPF (9, 10). Intestinal commensal microorganisms and their metabolites can impact distal lung tissues via immune, metabolic, and neuroendocrine pathways. Metagenomic/microbiome investigations and animal models demonstrate that dysbiosis aggravates bleomycin-induced pulmonary fibrosis, while microbial metabolites such as short-chain fatty acids exert anti-inflammatory and anti-fibrotic effects (9). Although data on the fecal microbiome in IPF are still scarce, an elevated pulmonary bacterial load during acute exacerbations is associated with unfavorable outcomes, indirectly suggesting the involvement of the gut ecosystem in disease progression. Furthermore, fecal microbiota transplantation from fibrosis-susceptible donors induces more severe fibrosis in germ-free mice, offering direct evidence of a causal relationship (11). Immune dysregulation is also central to IPF: an imbalance in alveolar macrophage M1–M2 polarization, activation of the Th17–IL-17 axis, and neutrophil oxidative burst sustain chronic inflammation and promote the release of pro-fibrotic mediators, including transforming growth factor-β (TGF-β), insulin-like growth factor-1 (IGF-1), and C-C motif chemokine ligand 18 (CCL18). Phenotypic alteration of regulatory T cells (Treg) may also cause them to shift from a protective to a pro-fibrotic role (12, 13). Despite the limited effectiveness of anti-inflammatory or immunosuppressive therapies, the dynamic equilibrium between immune cells and the microbiota remains a key regulatory point in disease progression.

**Figure 1:**
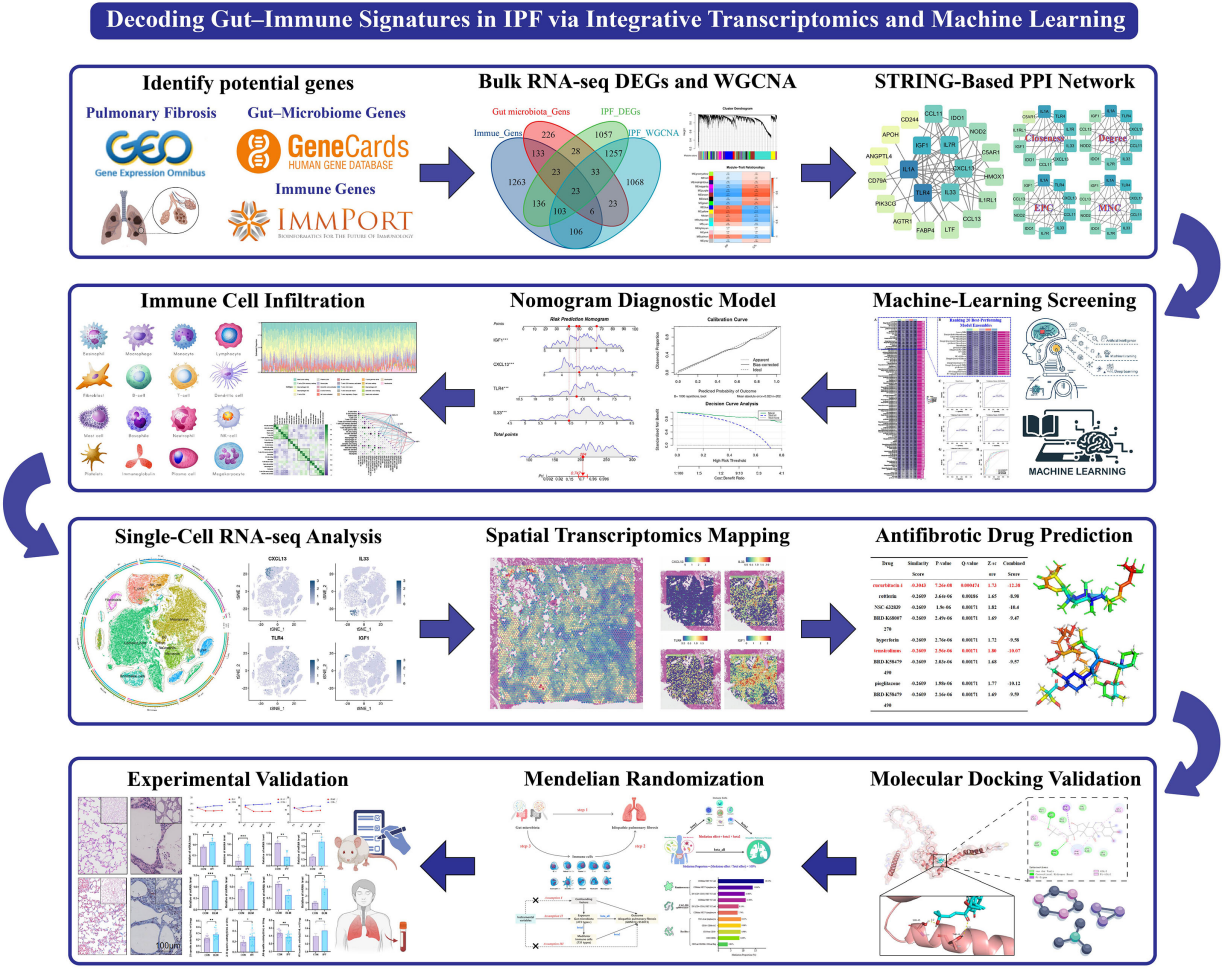
Study design overview Graphical overview of the study. Candidate genes were collated (GEO IPF cohorts, GeneCards gut-microbiome genes, and ImmPort immune genes), followed by bulk RNA-seq differential expression and WGCNA module detection; a STRING-based PPI network was built and hub genes prioritized. A 113-ensemble machine-learning screen identified core markers and supported a nomogram-based diagnostic model. Immune infiltration (CIBERSORT), single-cell RNA-seq, and spatial transcriptomics resolved cellular sources and spatial contexts. Drug candidates were predicted by signature reversal and evaluated by docking. Causal directionality and mediation along the “gut microbiome → immune phenotypes → IPF” axis were tested by two-sample Mendelian randomization; key targets were validated *in vitro*, *in vivo*, and in clinical samples. IPF, idiopathic pulmonary fibrosis; GEO, Gene Expression Omnibus; WGCNA, weighted gene co-expression network analysis; PPI, protein–protein interaction; MR, Mendelian randomization. ***P < 0.001.

These lines of evidence suggest that the pathogenesis of IPF involves multi-layer regulation within a “gut–immune–lung” network. To date, previous studies have mainly been restricted to single-omics analyses, lacking systematic integration and spatiotemporal validation. In this study, we aim to establish an integrative, cross-scale framework to characterize gut microbiota–host immune interactions in idiopathic pulmonary fibrosis (IPF) by combining bulk RNA-sequencing, single-cell transcriptomics, spatial transcriptomics, and peripheral-blood multi-omics. Specifically, we (i) integrate multi-omics evidence to map immune and stromal programs linked to fibrosis, (ii) apply machine-learning–based feature selection together with drug signature reversal prediction, molecular docking, and Mendelian randomization to prioritize key pathways and candidate targets, and (iii) validate selected targets using *in vitro* and *in vivo* experiments. Through this workflow, we seek to clarify how the gut–immune axis may contribute to IPF initiation and progression and to provide a reproducible roadmap for biomarker discovery and therapeutic target prioritization (**Figure 1**).

## 2 Materials and methods

### 2.1 Data acquisition and workflow

Multiple open-access transcriptomic resources were employed. At the bulk transcriptomic level, six Gene Expression Omnibus (GEO, RRID: SCR_005012) lung tissue cohorts were incorporated: GSE32537 (comprising 119 cases of IPF and 50 controls), GSE110147 (22 IPF cases and 11 controls), GSE150910 (103 IPF cases and 103 controls), GSE213001 (20 IPF cases and 14 controls), GSE53845 (40 IPF cases and 8 controls), and GSE92592 (20 IPF cases and 19 controls). These cohorts offered gene - expression profiles across a variety of IPF populations for the purposes of integration and model verification. To ensure methodological transparency, we additionally summarized the transcriptomic platforms: GSE32537 and GSE110147 were generated on the same microarray platform (GPL6244, Affymetrix Human Gene 1.0 ST Array), whereas GSE53845 (GPL6480, Agilent-014850 Whole Human Genome Microarray 4×44K), GSE92592 (GPL11154, Illumina HiSeq 2000), GSE213001 (GPL21290, Illumina HiSeq 3000), and GSE150910 (GPL24676, Illumina NovaSeq 6000) were profiled using distinct platforms. Accordingly, GSE32537 and GSE110147 were selected for cross-cohort merging and discovery-stage analyses (differential analysis and WGCNA) due to their shared platform, which enables more reliable batch-effect correction, while the remaining four heterogeneous-platform cohorts were retained as independent external validation datasets to assess model generalizability and mitigate overfitting. Regarding single-cell data, GSE122960 was analyzed, which encompasses single-cell RNA sequencing (scRNA-seq) data of the lungs from 4 IPF patients and 8 healthy controls. For spatial transcriptomics, GSE248082 human lung sections were utilized, including samples from 5 IPF cases (GSM7905763, GSM7905764, GSM7905765, GSM7905767, GSM7905768) and 5 controls (GSM8087031, GSM8087032, GSM8087033, GSM8087034, GSM8087035). All data were obtained from public databases, where the original acquisition had received ethics approvals. Our utilization of these data adhered to the terms of the repositories.

To systematically identify and validate gut–immune–linked molecular signatures in IPF, we established a step-by-step cross-scale workflow (**Supplementary Figure 4**). Briefly, we first acquired multi-layer open-access datasets, including bulk lung transcriptomes for discovery (GSE32537 and GSE110147) and independent external validation (GSE150910, GSE213001, GSE53845, and GSE92592), as well as scRNA-seq (GSE122960) and 10x Visium spatial transcriptomics (GSE248082). In parallel, GWAS summary statistics covering microbiome traits, immune traits, and IPF were collected for causal inference analyses, and orthogonal experimental materials (human plasma, bleomycin-induced mouse lungs, and TGF-β1–stimulated fibroblasts) were prepared for downstream validation. Next, bulk discovery cohorts underwent gene harmonization, normalization, and batch-effect correction (ComBat), with QC evaluation based on expression distribution and PCA. Candidate targets were then defined by integrating DEG signals (limma) and IPF-associated co-expression modules (WGCNA), followed by intersection with curated gut microbiota–related genes (GeneCards) and immune genes (ImmPort). These candidates were further prioritized through PPI-based hub screening (STRING/Cytoscape) and multi-algorithm machine-learning modeling, together with a core gene–based logistic nomogram with bootstrap internal validation. Model performance and biological interpretability were assessed using ROC/AUC in external cohorts and immune deconvolution (CIBERSORT) with gene–cell correlation analysis. Cross-scale validation was performed by localizing core genes to specific cell types and niches using scRNA-seq (Seurat/Harmony; SingleR; CellChat) and spatial mapping (Visium). Finally, drug repurposing was explored via L1000FWD coupled with molecular docking, and causal relationships along the gut–immune–lung axis were evaluated using two-sample Mendelian randomization and mediation analyses, followed by experimental validation in human, mouse, and cell models.

### 2.2 Identification of gut–immune–linked IPF target genes

We integrated the lung - expression datasets GSE32537 and GSE110147 (comparing IPF cases with controls) and performed a differential analysis based on the limma method, applying a false discovery rate (FDR) of less than 0.05 and an absolute fold - change greater than 1.3. Prior to downstream analyses, cross-cohort preprocessing was performed to reduce technical heterogeneity. Specifically, after gene-level harmonization and matrix merging, batch effects attributable to dataset origin were corrected using ComBat in the sva package (RRID: SCR_012836), with “dataset” specified as the batch variable (14, 15). To assess normalization and batch-correction quality, we generated boxplots of the standardized expression distributions across samples; consistent median alignment and comparable interquartile ranges across datasets were interpreted as successful normalization (**Supplementary Figure 2**). In addition, principal component analysis (PCA) was performed using FactoMineR/factoextra to visualize cohort separation before and after batch correction (16): **Supplementary Figure 3A** shows the PCA projection prior to batch removal (samples segregating by dataset), whereas **Supplementary Figure 3B** shows the PCA after ComBat correction (samples from different datasets becoming largely intermixed), supporting effective batch-effect mitigation. To further reduce noise from low-abundance transcripts, gene-wise mean expression was computed and ranked into percentiles; genes in the bottom 5th percentile by mean expression were removed prior to network construction and module detection.

Using the ClusterProfiler tool (RRID: SCR_016884) (17), we separately conducted enrichment analyses of the up - regulated and down - regulated gene lists for Gene Ontology (GO) Biological Processes and Kyoto Encyclopedia of Genes and Genomes (KEGG) pathways (with an adjusted P - value less than 0.05). To identify IPF - associated modules, we implemented weighted gene co - expression network analysis (WGCNA, RRID: SCR_003302) on the ComBat-corrected merged matrix (18). For computational efficiency and robustness, the top 5,000 most variable genes were selected according to median absolute deviation (MAD). Genes and samples were further quality-controlled using goodSamplesGenes; potential outlier samples were evaluated by hierarchical clustering (average linkage) and removed using a static cut height when applicable. Soft-thresholding power was determined using pickSoftThreshold over candidate powers; if an automatic estimate was not returned, an appropriate power was manually selected based on the evaluation of the “Scale-free topology fitting and mean connectivity for soft-threshold selection” plots. Network construction and module detection were performed using blockwiseModules with TOMType = “unsigned”, minModuleSize = 30, deepSplit = 2, and module merging threshold mergeCutHeight = 0.25. Modules that showed a significant positive correlation with the IPF phenotype were retained, and all genes within these modules were collected to define an “IPF co - expression core set.” Module–trait associations were quantified by Pearson correlation between module eigengenes and phenotype indicators, with corresponding Student asymptotic P-values reported in the module–trait heatmap. Subsequently, we retrieved “gut microbiota” protein-coding genes (n = 495) from GeneCards (RRID: SCR_002773) and human immune genes (n = 2,483) from ImmPort (RRID: SCR_012804) (19, 20). The intersections among the “IPF differentially expressed gene set,” “IPF core - module set,” “gut microbiota set,” and “immune gene set” were determined to identify “gut–immune–associated candidate targets for IPF” for subsequent downstream analyses.

### 2.3 PPI network construction and functional enrichment of core genes

Candidate genes were queried in the STRING database (RRID: SCR_005223) to acquire the protein–protein interaction (PPI) network (21), with the organism restricted to Homo sapiens and the interaction sources set to evidence-based channels (e.g., experiments, curated databases, co-expression, text mining, and neighborhood) according to STRING defaults. A medium confidence threshold was applied (minimum required interaction score), and isolated nodes were removed to ensure network interpretability. The resulting PPI network was subsequently visualized using Cytoscape (RRID: SCR_003032) (22). Leveraging the CytoHubba plugin (RRID: SCR_017677), genes were ranked according to four metrics, namely the maximum neighborhood component (MNC), degree centrality, edge percolated component (EPC), and closeness centrality. The top 10 genes from each metric were selected, and hub genes were defined through intersection (Venn-based overlap across the four ranking strategies), thereby reducing metric-specific bias. Enrichment analyses of the hub genes were conducted, including GO Biological Process and KEGG pathway enrichment using a consistent background gene universe, and multiple-testing correction was performed using the Benjamini–Hochberg method (adjusted P < 0.05). The enrichment networks were visualized using R packages such as ggplot2, igraph, and ggraph to generate term–gene and term–term relationship graphs, where edges represent shared genes and node sizes reflect gene counts or enrichment significance.

### 2.4 Integrated machine-learning framework for core-target selection

An integrated framework was constructed, which encompasses 12 algorithms, namely LDA, glmBoost, elastic net, random forest, GBM, SVM, LASSO, naïve Bayes, XGBoost, plsRglm, stepwise GLM, and ridge regression, resulting in 113 model combinations (23, 24). Through repeated cross - validation, the genes with the highest discriminative power were identified within the training set, and the robustness and generalizability were evaluated in an external validation set. To explicitly compare predictive performance across different algorithms/model combinations, the area under the receiver operating characteristic curve (AUC) was used as the primary evaluation metric. Specifically, for each model combination, ROC curves and AUC values were calculated in each resampling iteration of the repeated cross-validation, and the average AUC across iterations was used to rank and select the optimal model. The selected model was then further assessed in the external validation set, where AUC was again computed to confirm its generalizability.

### 2.5 Construction of a core gene–based clinical prediction model

A logistic model was constructed, with the expression of core genes serving as independent variables and the IPF status as the dependent variable. Here, “IPF status” refers to the binary case–control label defined by the original clinical diagnosis, where IPF patients were coded as 1 and healthy controls as 0. The model estimates the probability of being an IPF case based on gene-expression predictors (logit link). Leveraging the rms package, a nomogram was established to correlate expression values with risk scores and to estimate the probability for each patient. Internal validation was carried out through 1,000 bootstrap iterations. Calibration curves were plotted, and the mean absolute error was computed (25, 26). Additionally, decision - curve analysis was employed to assess the net benefit across a spectrum of clinical thresholds (27).

### 2.6 Immune cell infiltration analysis

Employing normalized bulk transcriptomes, we estimated the relative abundances of 22 immune cell types via CIBERSORT (RRID: SCR_016284) with 1,000 permutations (28). Specifically, the normalized expression matrix (genes × samples) was formatted to match the CIBERSORT input requirements, and the LM22 leukocyte signature matrix was used to infer immune-cell fractions. Samples were retained for downstream comparisons according to the CIBERSORT deconvolution significance (permutation-derived P value), ensuring robust estimates. The disparities between the IPF and control groups were examined using the Wilcoxon rank - sum test and presented visually through boxplots. Where applicable, P values were adjusted for multiple testing (Benjamini–Hochberg) across the 22 immune-cell types. Additionally, we calculated Spearman correlations between core genes and immune - cell fractions and constructed correlation networks to characterize the immune microenvironment of IPF and identify candidate key cell populations.

### 2.7 Single-cell transcriptomic analysis

The GSE122960 dataset was processed via a standardized scRNA-seq workflow employing Seurat (v4.3.0; v5-compatible workflow, RRID: SCR_016341) (29). Quality - control criteria were set as follows: the number of detected features ranged from 300 to 5,000, the total number of RNA counts was less than or equal to 20,000, the percentage of mitochondrial genes was less than or equal to 20%, and the percentage of hemoglobin genes was less than or equal to 1%. Through these criteria, 94,449 high - quality cells were obtained. The data were subjected to LogNormalize scaling. After normalization, the expression matrix was scaled prior to dimensionality reduction to ensure comparability across features. Subsequently, the top 3,000 highly variable genes were selected for principal component analysis (PCA), and batch effects were rectified using Harmony. Dimensionality reduction and downstream clustering were performed based on the leading principal components selected by standard variance/explained-variance diagnostics to balance signal preservation and noise reduction. Clustering was then carried out using the FindClusters functions with a resolution of 0.2. The cell distributions in IPF samples and control samples were visualized using t - SNE and UMAP. Cell annotation integrated both manual and automated methods. Cluster - specific markers were identified using the FindAllMarkers function and mapped to canonical markers for manual naming (30). Subsequently, automated labeling based on SingleR was performed on reference datasets. Cross - validation was employed to ensure the accuracy of cell annotation. The composition of cell types was compared between different groups, and the expression changes of core genes across clusters and groups were evaluated. DEGs were subjected to GO enrichment analysis to explore functional alterations. Using CellChat (RRID: SCR_023814), ligand - receptor networks were reconstructed to delineate active pathways, key interacting pairs, and communication centrality in IPF, emphasizing the crosstalk between immune and parenchymal cells and the potential mechanisms of core genes (31, 32). In CellChat analyses, interactions were inferred using the curated human ligand–receptor database, low-confidence pairs were filtered by minimum cell/spot counts and expression thresholds, and pathway-level aggregation was used to highlight dominant signaling programs.

### 2.8 Spatial transcriptomics analysis

The 10x Visium data from GSE248082 were analyzed using Seurat (v4.3.0; v5-compatible workflow). Following the loading of tissue sections and their registration to the corresponding images, SCTransform was implemented on a per - sample basis to normalize the sequencing depth. Spots failing standard quality criteria (e.g., extremely low UMI/features) were handled consistently across samples to reduce technical artifacts. Subsequently, highly variable genes were selected for PCA and clustering. The UMAP algorithm was employed to project spatial spots into a two-dimensional space, which was then mapped back onto the tissue to generate spatial cluster maps (33). Marker genes were identified via the FindAllMarkers function, and cell types were inferred using the SingleR algorithm to delineate fibroblast, epithelial, and immune - infiltrated regions. Spatial cluster annotations were further cross-checked against canonical marker expression to ensure biological plausibility in tissue context. The spatial architectures were quantitatively analyzed and compared between IPF samples and control samples. Spatially variable genes (SVGs) were identified using Moran’s I and related metrics, with a particular focus on the spatial expression of core genes (34). SVG detection was performed under a unified statistical framework, with multiple-testing correction applied where appropriate, and core-gene spatial patterns were evaluated both by spot-level expression gradients and by enrichment within disease-relevant regions (e.g., fibrotic foci-like areas). Spatial heatmaps were generated to illustrate their enrichment patterns and expression intensities across IPF and control sections, offering intuitive evidence regarding the spatiotemporal characteristics of core genes in IPF.

### 2.9 Screening candidate anti-fibrotic compounds

To identify potential therapeutic agents targeting core genes, an inquiry was made on the L1000 Fireworks Display (L1000FWD) platform to screen small molecules predicted to reverse the expression signature associated with IPF. The L1000FWD platform aggregates gene - expression profiles induced by compound perturbations, facilitating the detection of agents that are negatively correlated with the input gene pattern (35). The “gut–immune–IPF - related DEGs” were inputted, and the “reverse signature” mode was selected. Specifically, DEGs were separated into up-regulated and down-regulated lists using the predefined statistical thresholds described above, and gene identifiers were standardized to match the L1000FWD input format. The platform calculates the correlations between drug - induced expression shifts and the query gene set, resulting in a Combined Score and a Z - score. Candidate compounds were prioritized by jointly considering Combined Score and Z-score rankings, and the top-ranking hits were carried forward as a shortlist. Compounds ranked highly according to these metrics were further evaluated for their pharmacological properties and safety based on the existing literature to compile a shortlist of putative antifibrotic agents for downstream functional testing. This manual curation considered mechanism-of-action plausibility, reported antifibrotic/anti-inflammatory evidence, and feasibility for experimental validation.

### 2.10 Molecular docking to validate candidate drug–target binding

Molecular docking was employed to evaluate the interaction intensity and stability between candidates and core target proteins. The 3D structures of the targets were obtained from AlphaFold (RRID: SCR_018508) (AlphaFold Protein Structure Database; canonical human protein models were used as structural references, and model confidence was inspected using AlphaFold-provided per-residue confidence scores to ensure overall structural reliability in the docking regions) and pre - processed in PyMOL (RRID: SCR_000305), which involved the removal of waters, cofactors, and ligands, the completion of missing side chains, and the addition of polar hydrogens and Gasteiger charges (36). For targets with multiple domains and/or flexible regions, docking was performed using the biologically relevant structured region encompassing the putative binding pocket, while low-confidence/disordered segments were not used to define docking sites. The two-dimensional structures of the candidate compounds were obtained from PubChem (RRID: SCR_004284), followed by their protonation and charging processes. In AutoDock, grid boxes were positioned to encompass the putative active site and its adjacent regions. The docking box was centered on the reported/putative functional pocket (based on prior structural/functional knowledge where available), and its dimensions were set to fully cover the pocket and neighboring residues to avoid constraining ligand exploration. The docking process adopted a semi - flexible protocol with the following genetic algorithm parameters: a population size of 150, a maximum of 27,000 generations, and 10 runs. AutoDock Vina (RRID: SCR_011958) computations were used to estimate the binding energy (37). The results were ranked according to the binding energy, with lower values indicating stronger binding. The poses and interaction types were examined in PyMOL and Discovery Studio, and the occupancy of functionally crucial pockets and residues was verified. Complexes with energies below −5.0 kcal/mol and multiple stable contacts were considered favorable docking poses under this in silico scoring model. To assess robustness and protocol validity, we additionally implemented a validation strategy: (i) when an experimentally solved ligand-bound structure for a given target (or a closely related homolog/construct) was available, we performed redocking of the co-crystallized ligand into the same binding site and evaluated the agreement between predicted and reference poses using RMSD, with RMSD ≤ 2.0 Å considered a successful reproduction of the binding mode; (ii) for targets without suitable ligand-bound references, we evaluated internal consistency by repeating docking and confirming that top-ranked poses converged to similar binding orientations and key residue-contact patterns within the defined pocket. These checks provide empirical support for the reliability of the search configuration and the practical performance of the ranking for our target–ligand set.

### 2.11 Causal inference via Mendelian randomization

We employed two-sample Mendelian randomization (MR) to evaluate causal relationships and potential mediation among the gut microbiome, immune phenotypes, and IPF. MR uses genetic variants as instrumental variables (IVs) to infer causal effects of exposures on outcomes under random allocation principles, mitigating confounding and reverse causation. Analyses proceeded in three stages: (1) causal effects of gut microbiome taxa on IPF risk; (2) causal effects of immune cell-related phenotypes on IPF; and (3) multistep mediation models of “microbiome → immune phenotype → IPF” for taxa–immune pairs showing significant associations in steps 1–2. GWAS sources included summary statistics from European cohorts covering 211 gut microbiome taxa and 473 taxa classifications (38); GWAS of 731 peripheral-blood immune cell counts/proportions (39); and GBMI IPF GWAS data (1,028 cases and ~200,000 controls) (40, 41). All datasets comprised publicly available summaries without individual-level identifiers. IV selection used P < 1×10^-5^, with LD clumping (r² < 0.001, 10,000-kb window) to ensure independence; F statistics (F = β²/SE²) were computed to exclude weak instruments (F < 10). Reverse-causation testing applied a threshold of P < 5×10^-5^. Causal estimates were obtained primarily via inverse-variance weighting (IVW), with MR-Egger and weighted median as sensitivity analyses. Heterogeneity was assessed by Cochran’s Q; when significant, random-effects IVW was reported. Horizontal pleiotropy and outliers were evaluated with MR-PRESSO, and leave-one-out analyses assessed influence of individual IVs (42, 43). Effects are presented as odds ratios (ORs) with 95% CIs; P < 0.05 denoted significance. Mediation for significant microbiome–immune pairs was quantified as β_med = β_1_×β_2_ (β_1_: microbiome → immune; β_2_: immune → IPF), direct effect β_dir = β_all − β_med, and proportion mediated = β_med/β_all × 100%. Mediation was declared only when β_1_ and β_2_ were both significant and directionally concordant. CIs were computed via the delta method. All analyses were conducted in R using TwoSampleMR, MRPRESSO, and related packages.

### 2.12 *In vitro* and *in vivo* validation of core genes

To investigate the expression dynamics of key target genes in pulmonary fibrosis, we conducted experiments at the cellular, animal, and clinical levels. Human plasma samples were collected from 14 healthy volunteers and 14 patients diagnosed with IPF at General Hospital of Ningxia Medical University, with written informed consent and institutional approval (KYLL-2023-0516). IPF diagnosis was confirmed based on clinical history, physical examination, pulmonary function testing, high-resolution computed tomography, and lung biopsy, in accordance with the American Thoracic Society/European Respiratory Society guidelines. Plasma levels of IL-33, CXCL13, IGF-1, and TLR4 were measured using ELISA kits (YuanJu Institute of Biotechnology, China; catalog nos. YJ26377, YJ057504, YJ23240, JY027583). For *in vivo* validation, male C57BL/6J mice (22 ± 2 g, 6–8 weeks old, RRID: IMSR_JAX:000664) were intratracheally instilled with bleomycin sulfate (4 mg/kg in 30 µL of 0.9% NaCl) to establish a bleomycin-induced pulmonary fibrosis model. The mice were housed under specific-pathogen-free conditions with ad libitum access to water and standard chow. All mice were anesthetized by intraperitoneal injection of Avertin (tribromoethanol) at 12 mL/kg prior to the procedure. Adequate anesthesia was confirmed by generalized muscle relaxation and appropriate respiratory depth. After bleomycin administration, the animals were monitored daily for body weight and general condition. On day 21, mice were deeply anesthetized (Avertin [tribromoethanol], intraperitoneal injection, 12 mL/kg) and euthanized by CO_2_ inhalation in a euthanasia chamber. Compressed CO_2_ was introduced gradually and continuously through a pressure-reducing valve and flow meter at a displacement rate of 50% of the chamber volume per minute, and the chamber was not prefilled with CO_2_. After loss of consciousness and cessation of respiration, CO_2_ administration was continued for at least 5 min to ensure death. Death was confirmed by the absence of respiration, heartbeat, and reflexes before tissue collection, followed by cervical dislocation as a secondary physical method to ensure complete death. The animals were then placed in the supine position, and a rapid midline thoracotomy was performed to expose the heart and lungs. Lung tissues were immediately dissected, placed on ice, and the lung lobes were carefully separated using forceps for subsequent histopathological and biochemical analyses. The study adhered to institutional animal ethics guidelines (IACUC-NYLAC-2023-252). Concurrently, human lung fibroblasts (MRC-5, RRID: CVCL_0440) were cultured and stimulated with TGF-β1 (10 ng/mL) for 24 hours to induce fibroblast activation and myofibroblast differentiation. After harvesting, gene expression was analyzed by quantitative PCR (qPCR). For histopathological examination, lung tissues from the mouse models were fixed, embedded in paraffin, and sectioned. Hematoxylin-eosin and Masson’s trichrome staining were used to assess lung architecture and collagen deposition, respectively.

Total RNA from cells and lung tissues was isolated using TRIZOL reagent (Ambion), and cDNA was synthesized for qPCR analysis. To comply with MIQE recommendations (44), RNA quantity and purity were assessed spectrophotometrically, and only RNA samples meeting the predefined quality thresholds were used for reverse transcription and qPCR (A260/A280 = 1.90–2.00; A260/A230 = 2.00–2.20). No-template controls and reverse-transcription minus controls were included to monitor reagent contamination and residual genomic DNA amplification. β-actin was used as reference gene, and gene expression levels were calculated using the 2^-^ΔΔCt method. Reference-gene suitability was verified by confirming stable Ct distributions across experimental conditions (control vs. TGF-β1–treated fibroblasts; saline vs. bleomycin lungs), and amplification specificity was assessed by single-peak melt-curve profiles and the absence of non-specific bands/peaks where applicable. Primer sequences are provided in **Supplementary Table 13**, and primer performance was evaluated by standard-curve analysis using serially diluted cDNA to estimate amplification efficiencies; only assays showing acceptable efficiency and linearity were included for downstream quantification. For normalization, ΔCt values were obtained by subtracting β-actin Ct from target Ct within each sample, and relative expression was computed as 2^-^ΔΔCt using the matched control group as calibrator, with technical replicates averaged prior to statistical testing. Statistical analyses were performed using GraphPad Prism 9.5 (RRID: SCR_002798), with two-tailed Student’s t-test for two-group comparisons and one-way ANOVA for multiple-group comparisons. P-values of <0.05, <0.01, and <0.0001 were considered statistically significant.

### 2.13 Software and computational tools used in this study

All bioinformatic and statistical analyses were conducted primarily in R (R Foundation for Statistical Computing, RRID: SCR_001905) using reproducible scripts and, where applicable, R Markdown/knitr for report generation. Public transcriptomic datasets were obtained from the NCBI Gene Expression Omnibus using GEOquery and the tinyarray toolkit. Candidate gut–immune–linked targets were curated from the GeneCards and ImmPort databases. Gene identifier harmonization and annotation were performed with org.Hs.eg.db, supported by general data handling/utility packages including stringr, dplyr, tidyverse, data.table, and file I/O packages such as openxlsx and writexl.

Differential expression analyses and visualization were dependent on the functions implemented in the tinyarray R package, in conjunction with standard plotting packages (ggplot2, RRID: SCR_014601; ggthemes, enrichplot) and auxiliary visualization packages (pheatmap, ComplexHeatmap, RColorBrewer). Co-expression network analysis was performed using WGCNA, with additional plotting support from gplots. Functional enrichment analyses were carried out using clusterProfiler (with org.Hs.eg.db as the annotation reference), and results were visualized using ggplot2/enrichplot and related packages. Protein–protein interaction networks were constructed using the STRING database, followed by network visualization and hub-gene prioritization in Cytoscape with the cytoHubba plugin; graph-based plotting and network handling in R additionally employed igraph and ggraph.

The integrated machine-learning workflow for core-target selection was implemented in R using a combination of general modeling utilities and algorithm-specific libraries, including caret (model training utilities), glmnet (LASSO, ridge regression, and elastic net), mboost (glmBoost), randomForestSRC (random forest), e1071 (SVM and naive Bayes), plsRglm (PLS-GLM), gbm (gradient boosting machine), xgboost (XGBoost), and MASS (e.g., LDA and stepwise model selection via stepAIC). Model evaluation and performance visualization used pROC (ROC/AUC), ComplexHeatmap (AUC heatmaps), and UpSetR (feature intersection). Custom utility functions and wrappers for model training, feature extraction, scaling, and evaluation were organized in internally maintained R scripts (e.g., a dedicated “pipline.Machine.Learning.R” module) to ensure consistent processing across cohorts. To facilitate reproducibility, the detailed source code for this machine-learning script has been deposited in a public GitHub repository (https://github.com/HTX2023/Multi-omics-MachineLearning-in-IPF) to allow for transparent review and application of the methodology.

Construction of the core gene–based clinical prediction model (including nomogram development, calibration, and decision-curve analysis) was performed using rms, regplot, and rmda, with additional validation support from ResourceSelection. Immune cell infiltration analysis was conducted using the CIBERSORT methodology with the LM22 signature matrix, implemented via CIBERSORT and dependency support (e.g., bseqsc), and visualized using ggplot2, pheatmap, ComplexHeatmap, ggpubr, corrplot, and psych; gene–immune association plots used utilities including ggExtra and network-style visualization with linkET and ggraph. Single-cell transcriptomic analyses were performed with Seurat (v4.3.0; v5-compatible workflow), batch correction via harmony, marker identification support via COSG, and visualization/plot assembly using patchwork, cowplot, and related tidyverse packages; automated cell-type annotation was performed using SingleR, reference resources from celldex (e.g., HumanPrimaryCellAtlas-based references), parallelization utilities from BiocParallel, and data structure conversion using SingleCellExperiment.

## 3 Results

### 3.1 Identification and functional profiling of DEGs in IPF

**Figure 2:**
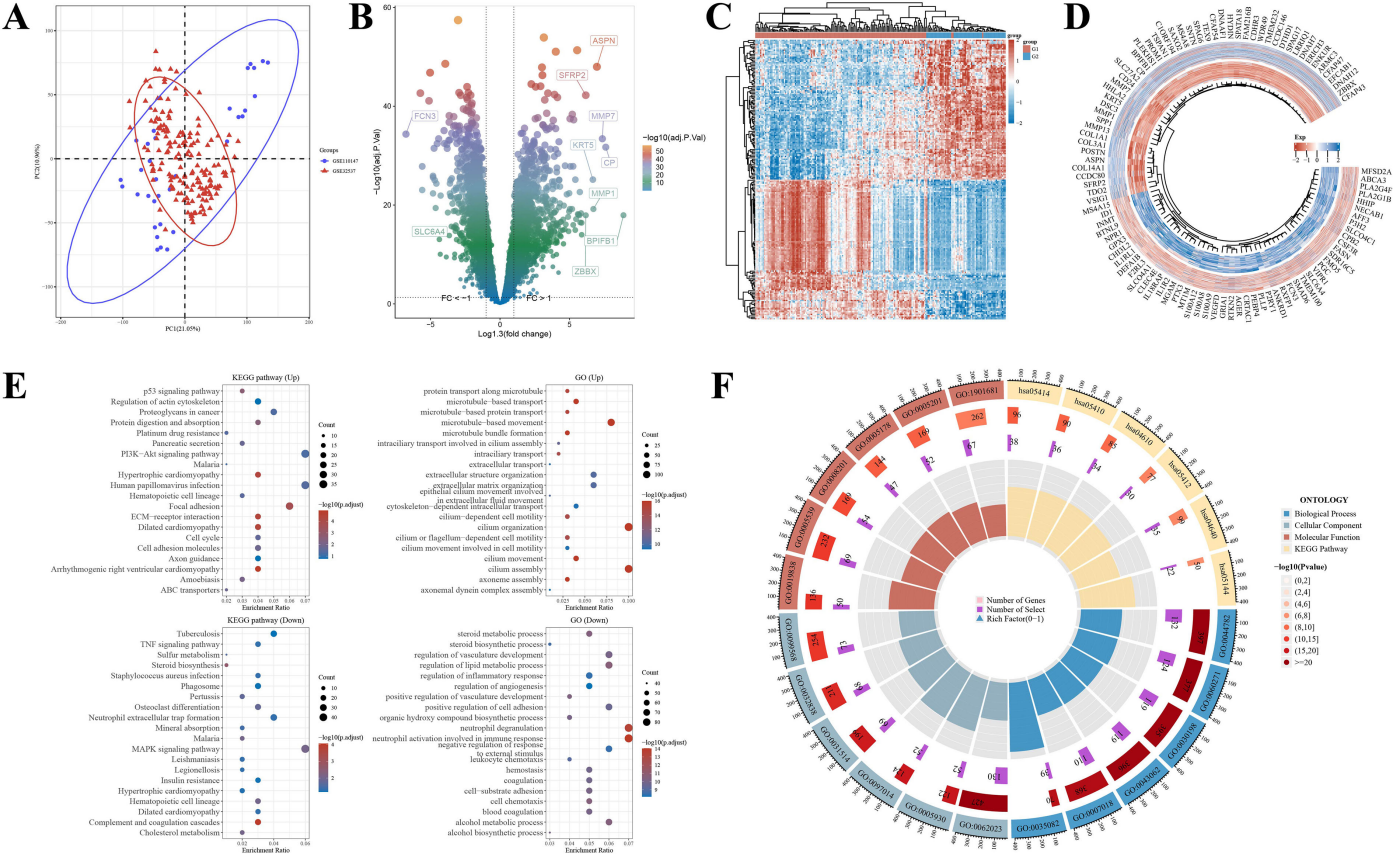
Differential expression landscape and functional enrichment in IPF lungs **(A)** Principal-component analysis showing separation between IPF and control samples in integrated cohorts. **(B)** Volcano plot of DEGs (|fold change| > 1.3, FDR < 0.05). **(C)** Heatmap of representative DEGs. **(D)** Circular heatmap of top dysregulated genes ranked by fold change. **(E)** GO/KEGG enrichment of up-regulated genes highlights ECM organization, cilium assembly, PI3K–AKT, and ECM–receptor interaction. **(F)** Integrated ring plot summarizing GO/KEGG terms for down-regulated genes (e.g., lipid/steroid metabolism, neutrophil-related regulation, MAPK and cholesterol pathways). DEG, differentially expressed gene; GO, Gene Ontology; KEGG, Kyoto Encyclopedia of Genes and Genomes; ECM, extracellular matrix; FDR, false discovery rate.

In the integrated cohorts GSE32537 and GSE110147, applying thresholds of |fold change| > 1.3 and FDR < 0.05, we identified 2,660 DEGs, with 1,355 being upregulated and 1,305 downregulated (**Figures 2A–D**; **Supplementary Table 1**). The upregulated genes were significantly enriched in GO biological processes such as microtubule-based transport, extracellular matrix (ECM) organization, epithelial cilium movement, and cilium assembly. Corresponding KEGG pathways included the p53 signaling pathway, PI3K–Akt signaling, ECM–receptor interaction, and cell adhesion molecules. Conversely, the downregulated genes were enriched in GO terms related to steroid metabolic processes, regulation of lipid metabolism, cell–matrix adhesion, and negative regulation of neutrophil activation involved in the immune response. The associated KEGG pathways included MAPK signaling, cholesterol metabolism, neutrophil extracellular trap (NET) formation, and TNF signaling (**Figures 2E–F**; **Supplementary Table 2**). These findings suggest a systemic disequilibrium in IPF lungs, characterized by the upregulation of structural remodeling programs alongside the downregulation of metabolic and inflammatory regulatory pathways.

### 3.2 Gene modules correlated with IPF phenotype and gut–immune interactions

**Figure 3:**
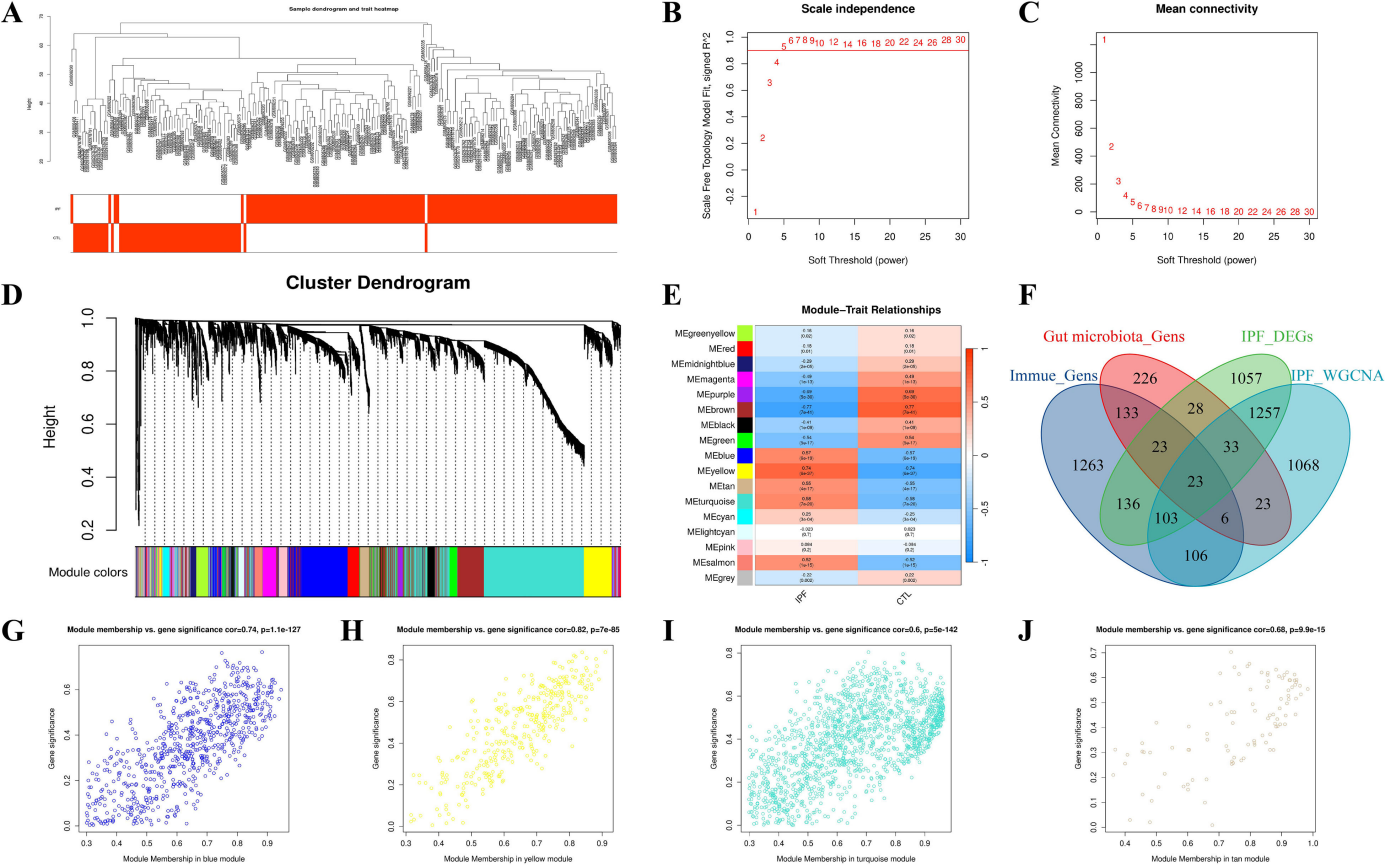
WGCNA identifies IPF-associated modules and gut–immune candidate genes **(A)** Sample clustering and trait heatmap. **(B, C)** Scale-free topology fitting and mean connectivity for soft-threshold selection (β = 5). **(D)** Gene dendrogram with dynamic tree cut module colors. **(E)** Module–trait correlations highlighting IPF-associated modules. **(F)** Four-way Venn integrating IPF DEGs, IPF WGCNA modules, immune-related genes, and gut-microbiota-related genes to yield 23 candidates. **(G–J)** Module membership versus gene significance scatterplots for representative IPF-associated modules (blue, yellow, turquoise, tan). MM, module membership; GS, gene significance.

Utilizing WGCNA with a soft threshold of β = 5, we identified 17 distinct modules (**Figures 3A–D**; **Supplementary Table 3**). Among these, the blue, yellow, turquoise, and tan modules exhibited a strong positive correlation with the IPF phenotype (P < 0.05) (**Figures 3E, G–J**). By intersecting the genes from these modules with the IPF DEG set, as well as immune-related and gut microbiota-related genes, we identified 23 candidate genes associated with the “gut-immune” axis in IPF (**Figure 3F**; **Supplementary Table 4**). These candidates form the core set for subsequent network analysis and feature selection.

### 3.3 PPI network analysis reveals key hub genes in IPF

**Figure 4:**
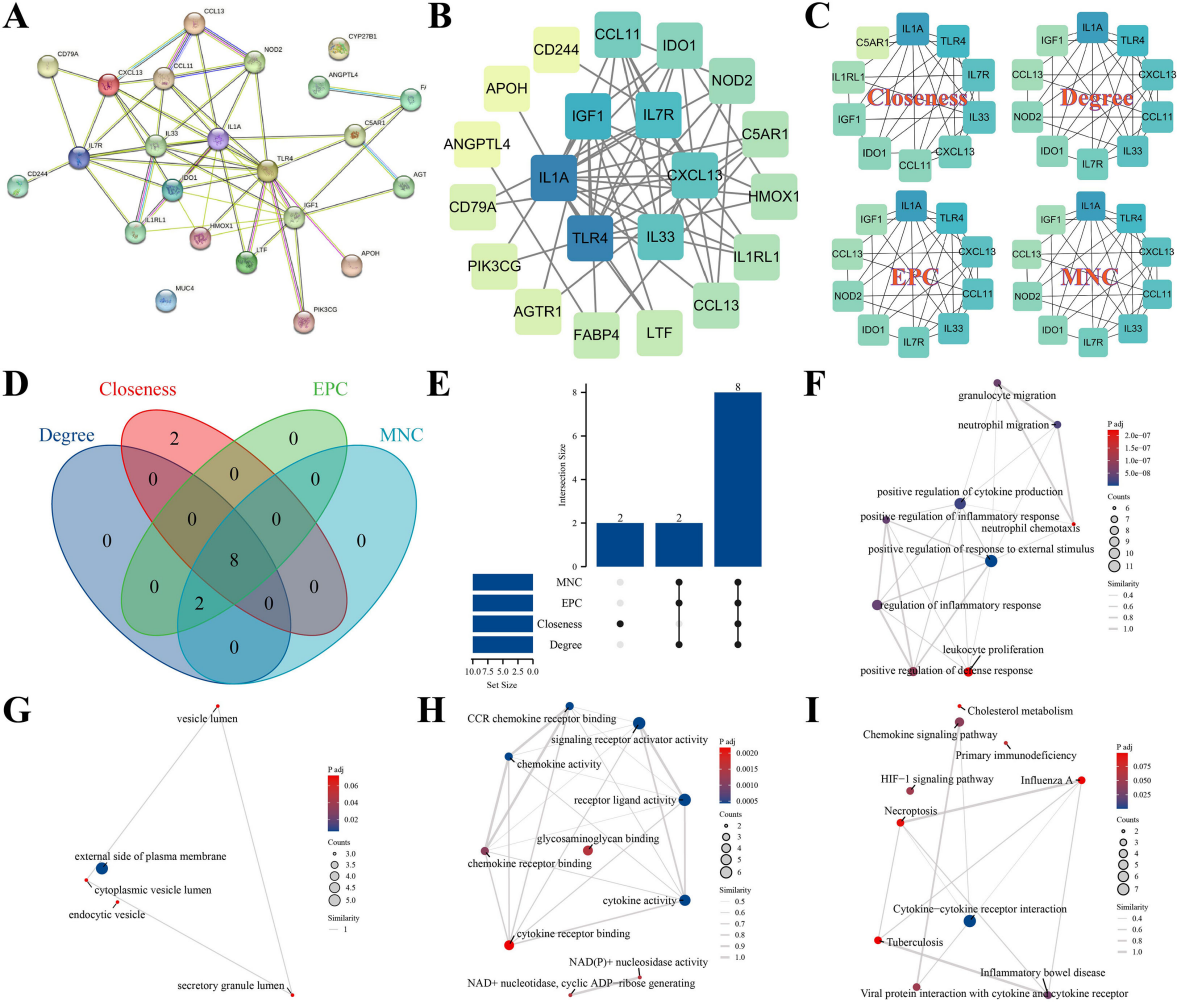
STRING-derived PPI network, hub selection, and functional attributes **(A) **Global PPI network of the 23 candidates. **(B)** Representative subnetwork centered on high-connectivity nodes. **(C)** Hub ranking visualization across four CytoHubba metrics (degree, MNC, EPC, closeness). **(D)** Venn diagram of top-10 lists across metrics; **(E)** intersection size plot identifying eight robust hubs. **(F–H)** Enrichment networks for GO biological process and molecular function (e.g., neutrophil migration, cytokine activity, CCR chemokine receptor binding). **(I)** KEGG pathway network emphasizing chemokine/cytokine–receptor signaling and cholesterol metabolism. STRING, Search Tool for the Retrieval of Interacting Genes/Proteins; MNC, maximum neighborhood component; EPC, edge percolated component.

A PPI network was constructed based on STRING data, encompassing 23 genes (**Figures 4A, B**; **Supplementary Table 5**). By applying four topological metrics—MNC, degree centrality, EPC, and closeness centrality—we identified the top 10 genes for each metric (**Figure 4C**). The intersection of these lists led to the identification of eight robust hub genes (**Figures 4D–E**). Enrichment analyses indicated that these hub genes were predominantly associated with biological processes such as neutrophil migration, positive regulation of cytokine production, and regulation of inflammatory response. In terms of cellular components, the hub genes were enriched in the cytoplasmic vesicle lumen and secretory granule lumen. Regarding molecular functions, the hub genes were involved in CCR chemokine receptor binding, chemokine activity, and cytokine activity. KEGG pathway analysis further underscored the involvement of these genes in cholesterol metabolism, chemokine signaling, and cytokine–receptor interactions, highlighting the critical roles of immune chemotaxis and lipid metabolism within the network (**Figures 4F–I**; **Supplementary Table 6**).

### 3.4 Machine learning-based gene selection and validation across independent cohorts

Among 113 model combinations encompassing 12 algorithms—namely LDA, glmBoost, elastic net, rand.

**Figure 5:**
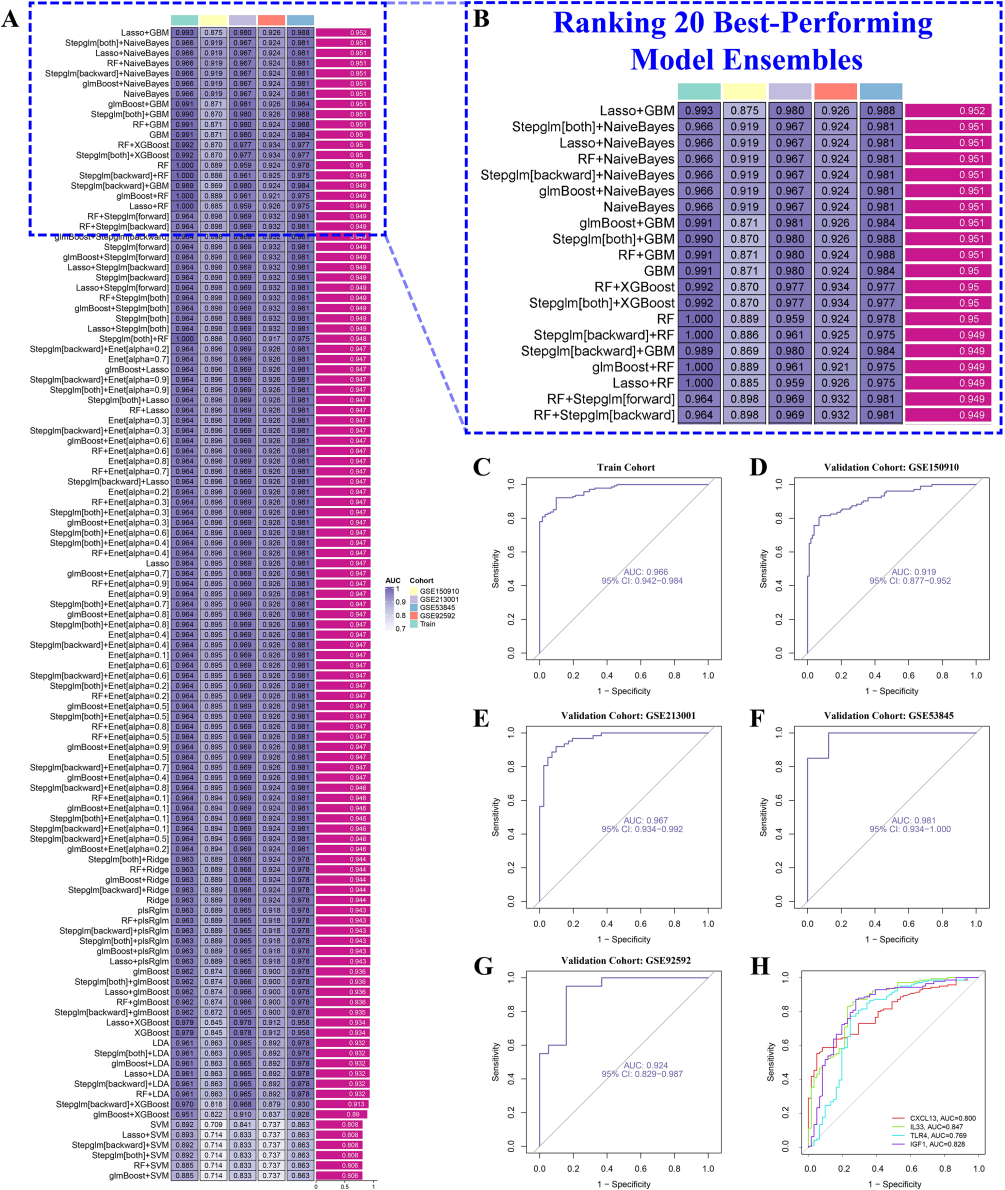
Integrated machine-learning screening and cross-cohort validation **(A)** Performance heatmap of 113 model ensembles across training/validation cohorts. **(B)** Top-20 ensembles ranked by AUC (e.g., LASSO+GBM, Stepglm[both]+NaiveBayes). **(C)** ROC curve in the training cohort (combined GSE32537+GSE110147). **(D–G)** ROC curves in independent validation cohorts (GSE150910, GSE213001, GSE53845, GSE92592). **(H)** Single-gene ROC performance for CXCL13, IL33, TLR4, and IGF1. Core markers consistently outperformed single genes and generalized across datasets. AUC, area under the ROC curve; GBM, gradient boosting machine; ROC, receiver-operating characteristic.

GBM, SVM, LASSO, naïve Bayes, XGBoost, plsRglm, stepwise GLM, and ridge regression—four diagnostic core markers, CXCL13, IL33, TLR4, and IGF1, were consistently selected (**Figures 5A, B**; **Supplementary Table 7**). The model trained on the combined dataset (GSE32537 + GSE110147) achieved an area under the curve (AUC) of 0.966 (**Figure 5C**). Across four independent validation cohorts (GSE150910, GSE213001, GSE53845, GSE92592), the mean AUC was 0.947 (**Figures 5D–G**). The performance of individual genes was as follows: CXCL13 with an AUC of 0.800, IL33 with an AUC of 0.847, TLR4 with an AUC of 0.769, and IGF1 with an AUC of 0.828 (**Figure 5H**). These results indicate that the multigene panel demonstrates superior discrimination and robust cross-cohort generalizability.

### 3.5 Development of a clinical prediction model using core genes in IPF

**Figure 6:**
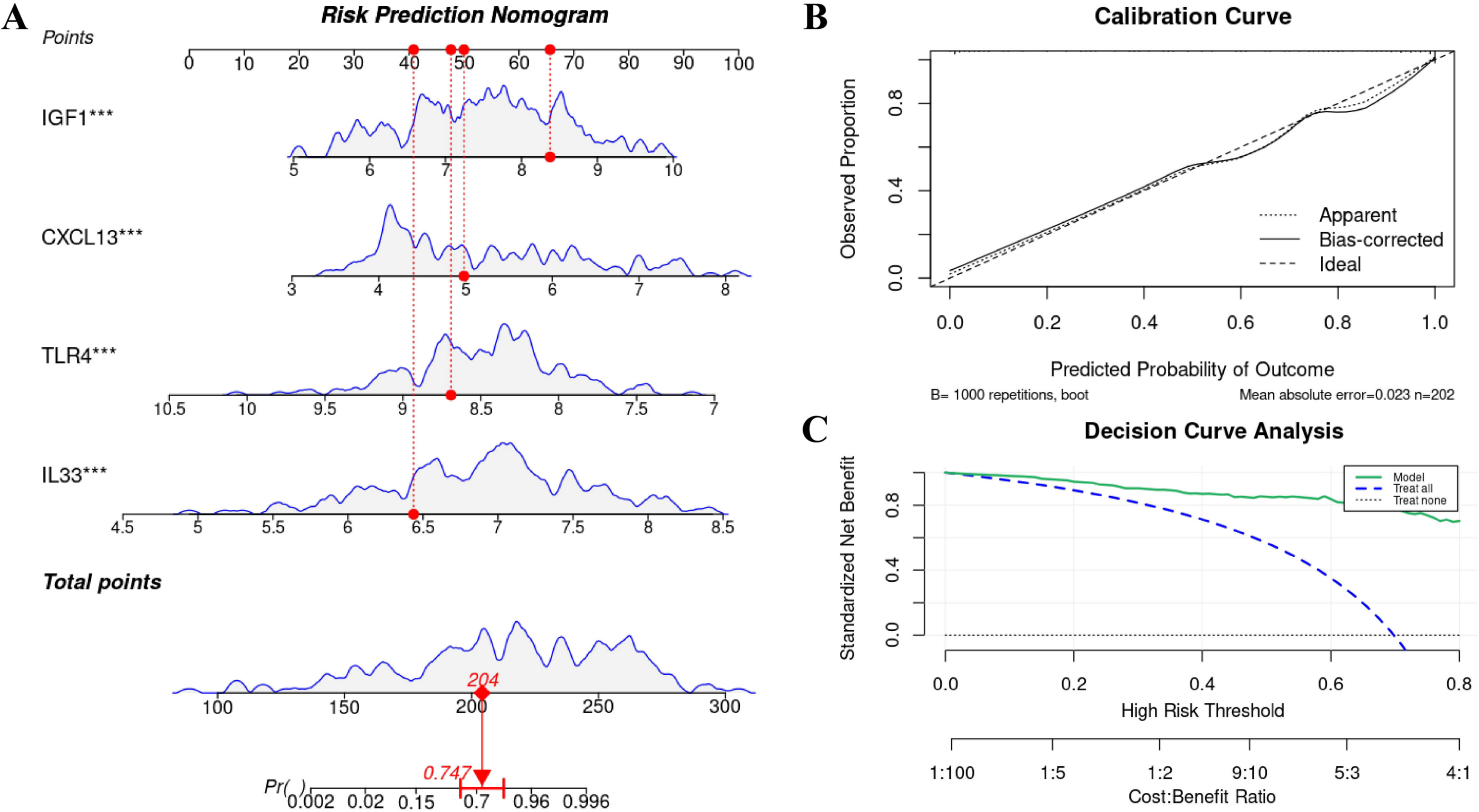
Nomogram-based diagnostic model and clinical utility assessment **(A)** Nomogram translating expression of CXCL13, IL33, TLR4, and IGF1 into individualized IPF risk. **(B)** Calibration curve after 1,000-bootstrap internal validation showing close agreement between predicted and observed probabilities (MAE reported). **(C)** Decision-curve analysis demonstrating higher standardized net benefit across wide threshold ranges versus “treat-all”/”treat-none” strategies. MAE, mean absolute error. *** denotes P < 0.001.

A nomogram constructed from the four core genes facilitated the quantification of individualized risk, with the total score consistently correlating with disease probability. For example, a total score of 204 was associated with an IPF probability of approximately 0.747 (**Figure 6A**). Internal validation using 1,000 bootstrap resamples revealed a strong concordance between the calibration curve and the ideal line, with a mean absolute error (MAE) of 0.023 (n = 202), indicating a high level of agreement between predicted probabilities and observed outcomes (**Figure 6B**). Decision-curve analysis indicated a consistently higher standardized net benefit compared to “treat-all” or “treat-none” strategies across a wide range of thresholds (**Figure 6C**), thereby supporting the model’s clinical utility.

### 3.6 Immune cell infiltration patterns and their correlation with core genes in IPF

**Figure 7:**
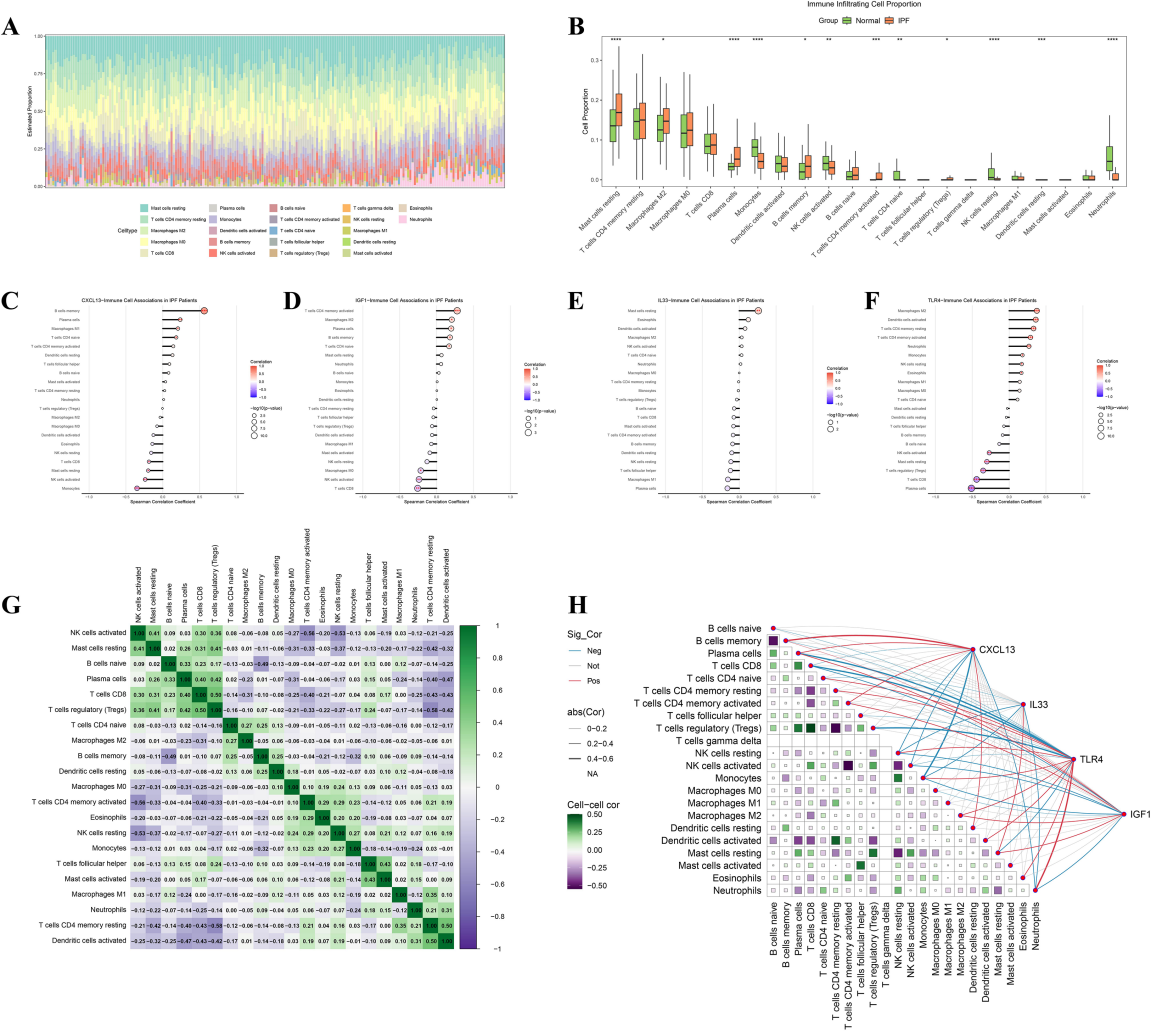
Immune-cell landscape by CIBERSORT and correlations with core genes **(A)** Stacked composition of 22 leukocyte subsets per sample. **(B)** Groupwise boxplots showing increased resting mast cells, M2 macrophages, plasma cells, memory B cells, and activated CD4 memory T cells in IPF, with reductions in monocytes, activated NK cells, and neutrophils. **(C–F)** Gene–cell association plots for CXCL13, IGF1, IL33, and TLR4 (Spearman coefficients, significance coded). **(G)** Pairwise correlation heatmap among immune subsets revealing coordinated pro-/anti-inflammatory patterns. **(H)** Bipartite network linking core genes to significantly associated immune populations. NK, natural killer. *P < 0.05, **P < 0.01, ***P < 0.001, and ****P < 0.0001.

The CIBERSORT deconvolution analysis indicated a significant increase in the abundance of resting mast cells, M2 macrophages, plasma cells, memory B cells, and activated CD4 memory T cells in IPF, accompanied by a decrease in monocytes, activated natural killer (NK) cells, and neutrophils (all P < 0.05) (**Figures 7A, B**; **Supplementary Table 8**). The core gene–immune cell correlations were as follows: CXCL13 demonstrated positive associations with memory B cells, plasma cells, M1 macrophages, and naïve CD4 T cells; IGF1 was positively associated with activated CD4 memory T cells, M2 macrophages, plasma cells, and memory B cells; IL33 showed a correlation with resting mast cells; and TLR4 was correlated with M2 macrophages, neutrophils, monocytes, and eosinophils (**Figures 7C–F, H**). The pairwise correlation network underscored systemic patterns of synergy and antagonism among the immune cell subsets (**Figure 7G**).

### 3.7 Single-cell RNA-seq analysis unveils differential expression and intercellular communication in IPF

**Figure 8:**
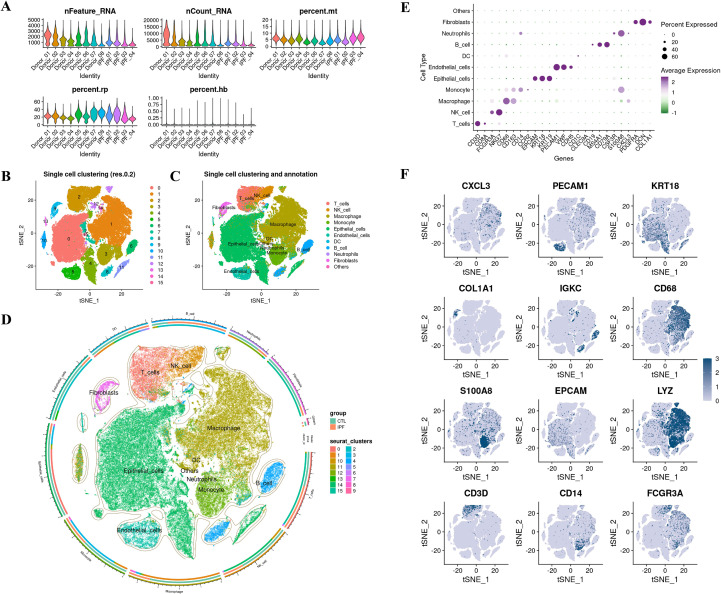
Single-cell quality control and atlas construction **(A)** QC violin plots for nFeature_RNA, nCount_RNA, mitochondrial/hemoglobin percentages after filtering. **(B)** t-SNE clustering (resolution = 0.2). **(C)** Manual/automated cell-type annotation showing major lineages (T, NK, macrophage/monocyte, epithelial, endothelial, dendritic, B, neutrophil, fibroblast, others). **(D)** Circular map summarizing cluster identities and group contributions (IPF vs control). **(E)** Dot plot of canonical markers across annotated lineages. **(F)** Feature plots for representative markers (e.g., PECAM1, KRT18, COL1A1, S100A8, EPCAM, LYZ, CD3D, CD14, FCGR3A). QC, quality control; t-SNE, t-distributed stochastic neighbor embedding.

**Figure 9:**
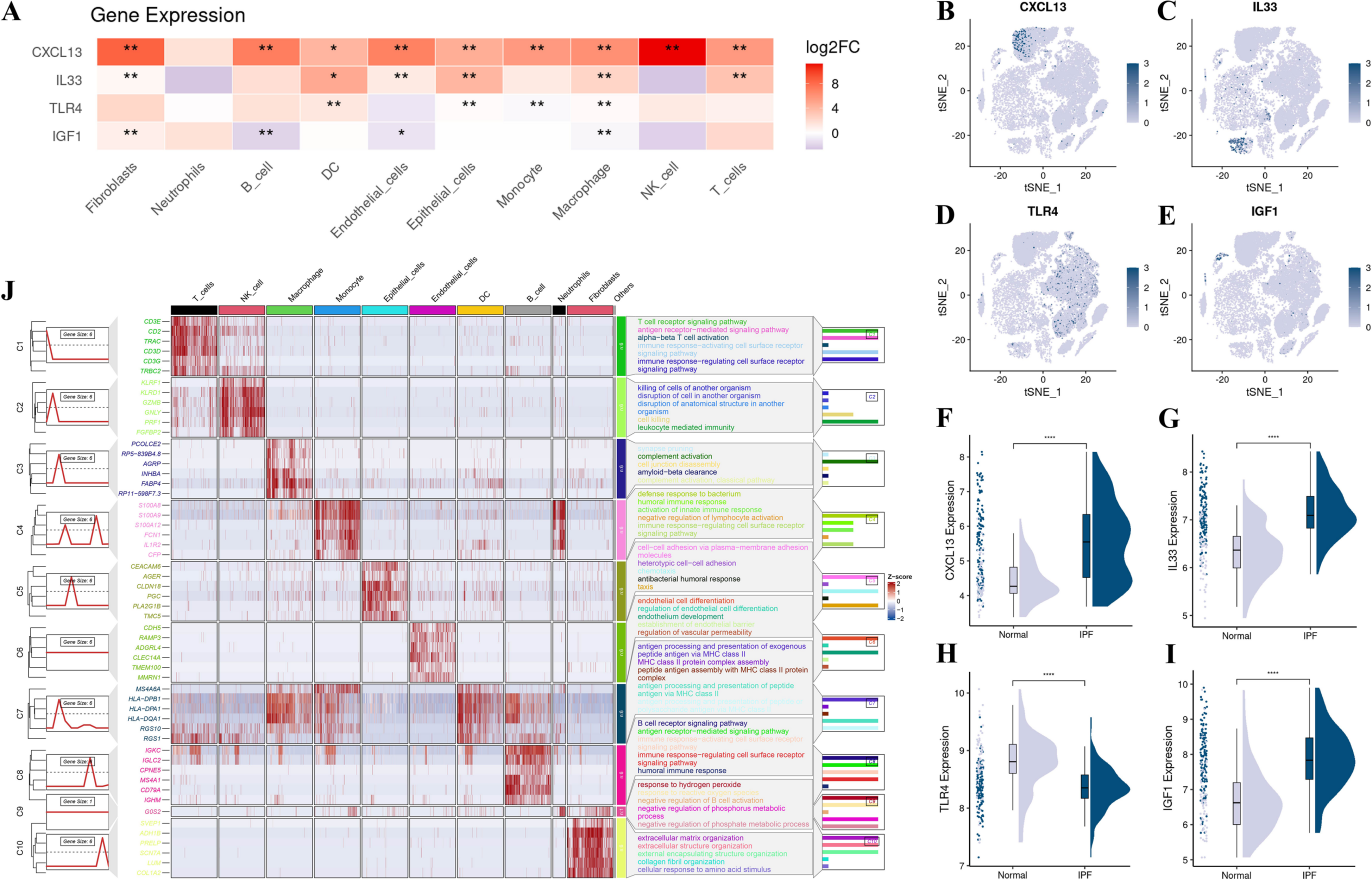
Single-cell distribution of core genes and functional programs **(A)** Heatmap of log2FC expression of CXCL13, IL33, TLR4, and IGF1 across major cell lineages. **(B–E)** Feature plots showing spatial distribution of each core gene across clusters. **(F–I)** Integrated bulk RNA-seq expression analysis comparing core gene expression in IPF and control groups. **(J)** GSVA-style heatmaps with pathway summaries highlighting immune-activation signatures and stromal programs in cell-type-specific patterns. *P < 0.05, **P < 0.01, and ****P < 0.0001.

**Figure 10:**
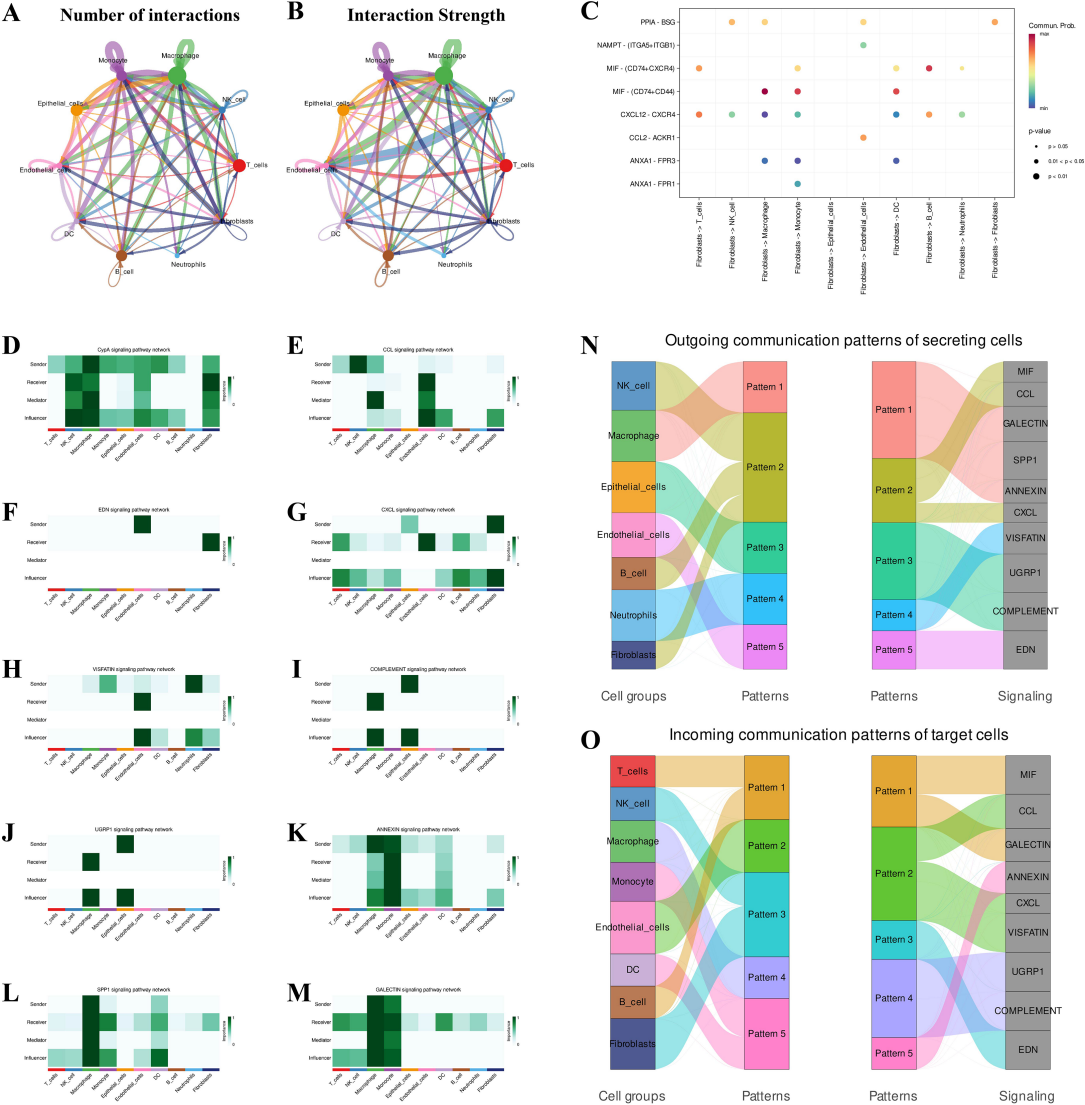
Cell–cell communication reveals fibroblast–innate immune crosstalk in IPF CellChat inference across annotated lineages. **(A)** Network graph of number of interactions; **(B)** interaction strength. **(C)** Bubble plot of significant ligand–receptor pairs (e.g., MIF–CD74/CD44, CXCL family). **(D–M)** Pathway-specific sender/receiver heatmaps for representative signals (e.g., CCL/CCR, EDN, VGF/NGF, LCP1, ANNEXIN, GALECTIN, SPP1). **(N)** Sankey diagram summarizing outgoing communication patterns by sender cell groups. **(O)** Sankey diagram of incoming communication patterns in target cell groups. Fibroblasts emerge as dominant senders within CXCL/MIF networks, while endothelial cells and macrophage/monocyte lineages act as major receivers, consistent with enhanced immune–stromal coupling in IPF. LR, ligand–receptor.

Following rigorous QC and annotation, we resolved 11 clusters: T cells, NK cells, macrophages, monocytes, epithelial cells, endothelial cells, dendritic cells, B cells, neutrophils, fibroblasts, and Others (**Figure 8**). Between-group analysis showed CXCL13 upregulation across many lineages—most striking in fibroblasts and NK cells; IL33 increased in fibroblasts, endothelium, epithelium, and macrophages; TLR4 exhibited cell-type-specific upregulation in dendritic cells, macrophages, and epithelial cells. Despite the overall downregulation at the bulk level, this phenomenon likely reflects compositional shifts and niche-specific activation; IGF1 increased in fibroblasts but decreased in B cells, endothelial cells, and macrophages (**Figures 9A–E**; **Supplementary Table 9**). In the integrated bulk RNA-seq expression data analysis, both the IPF and control groups were assessed. The results indicated that CXCL13, IL-33, and IGF-1 were significantly higher in the IPF group compared to the control, while TLR4 expression was significantly lower in the IPF group (**Figures 9F–I**). Fibroblast differential expression profiles demonstrated significant enrichment in ECM organization, extracellular structure assembly, and collagen fiber remodeling (**Figure 9J**). Ligand–receptor inference identified maximal communication strength between fibroblasts and macrophages/monocytes (**Figures 10A, B**). Fibroblast-to–innate immune signaling was dominated by the MIF–(CD74 + CD44) axis (**Figure 10C**); fibroblasts functioned as principal senders/influencers and endothelial cells as principal receivers within CXCL and MIF networks. Fibroblast incoming signaling was enriched for CypA and EDN pathways (**Figures 10D–M**). Pattern analysis indicated macrophages remained active through GALECTIN, SPP1, and ANNEXIN networks; fibroblasts preferred pattern 3 for incoming (e.g., EDN) and pattern 2 for outgoing (e.g., CXCL/MIF) communications, with enhanced cross-lineage links to B and NK cells (**Figures 10N–O**).

### 3.8 Spatial transcriptomic mapping highlights fibroblast-enriched regions in IPF tissues

**Figure 11:**
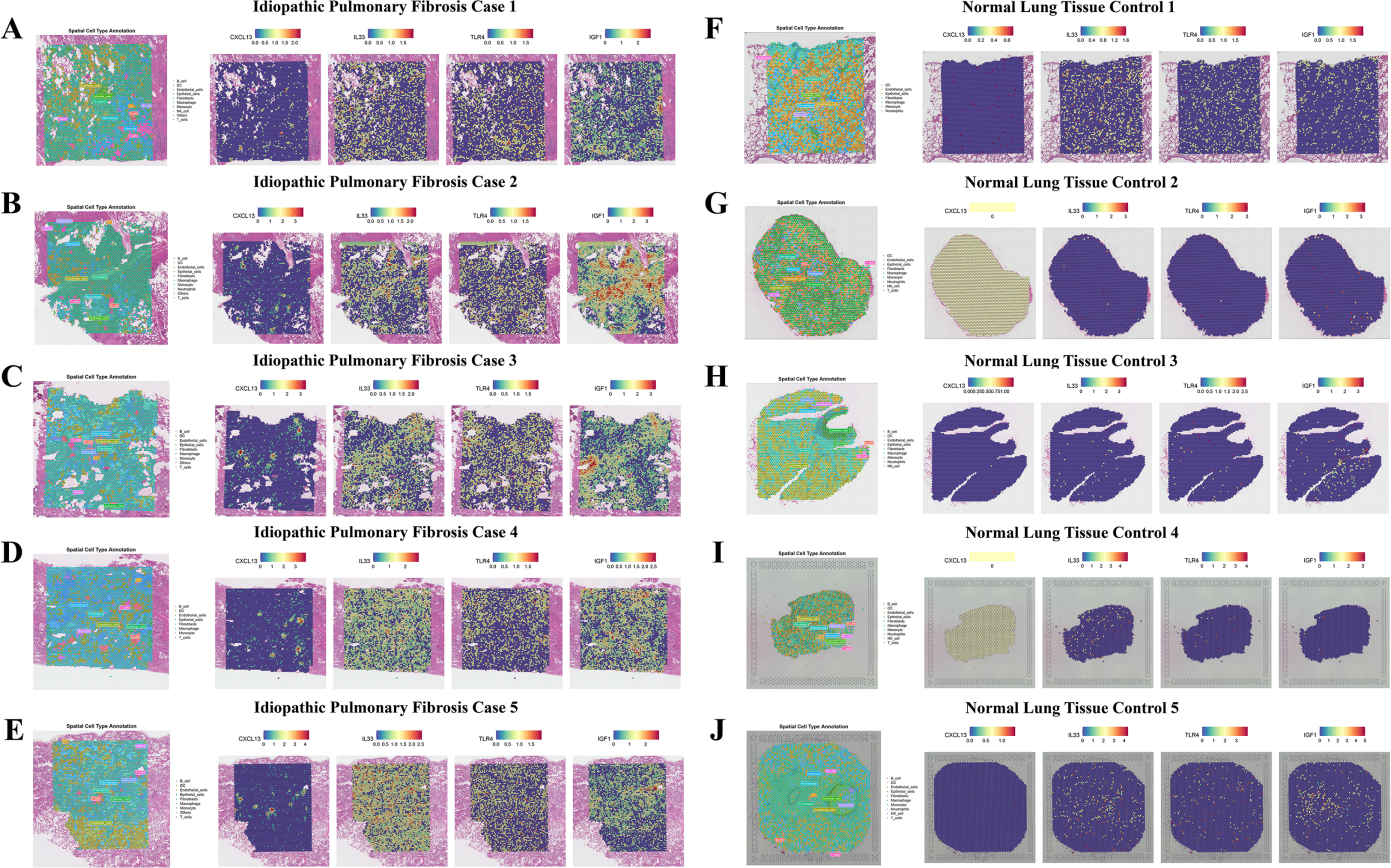
Spatial transcriptomic mapping of core genes in IPF and controls Ten 10x Visium lung sections from GSE248082 are shown. **(A–E)**, IPF cases 1–5; **(F–J)**, normal controls 1–5. For each specimen, the leftmost panel shows spatial cell-type annotation (Seurat inference overlaid on H&E), and the four right panels display spot-level normalized expression of CXCL13, IL33, TLR4, and IGF1 (color bars denote relative expression from low to high). IPF tissues exhibit expanded fibroblast-rich territories spatially interdigitating with epithelial and macrophage regions, accompanied by higher IL33 and IGF1 signals and modest TLR4 upregulation; CXCL13 is focal but increased relative to controls. Control lungs show predominantly epithelial/endothelial annotations with uniformly low expression of all four genes. H&E, hematoxylin–eosin.

In spatial analyses of tissue sections from five IPF cases and five control samples, fibroblasts were found to occupy the largest area fraction within IPF tissue (**Figures 11A–E**). These fibroblasts demonstrated significant proximity to epithelial cells and macrophages, indicating a spatial coupling among epithelial, mesenchymal, and immune components within fibrotic foci. The spatial expression of key genes revealed that IL33 and IGF1 were significantly upregulated in IPF tissues compared to controls, whereas TLR4 displayed a heterogeneous, niche-dependent pattern with focal enrichment in immune-infiltrated regions despite an overall reduction at the bulk level. Although CXCL13 expression was lower than that of the aforementioned genes, it was still higher than in control samples (**Figures 11F–J**). The overall spatial architecture observed was consistent with findings from single-cell expression and intercellular communication analyses.

### 3.9 Identification of antifibrotic drug candidates through gene signature reversal

**Figure 12:**
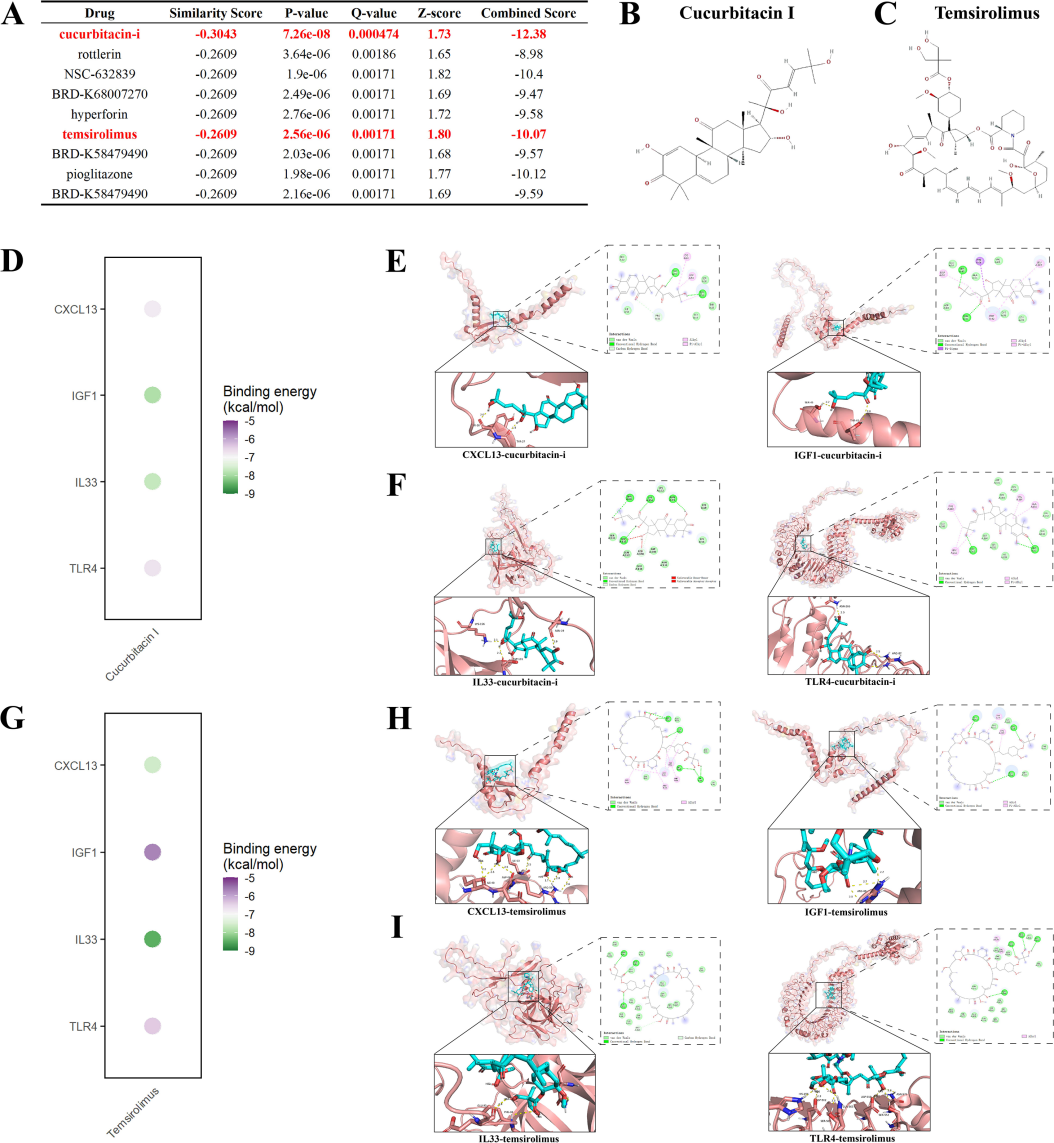
In-silico drug prioritization and docking to core targets **(A)** Top candidates from L1000FWD reverse-signature screening using gut–immune–IPF DEGs (up and down sets queried separately). Cucurbitacin I and temsirolimus rank consistently among the most negative (strongest reversal) by Combined Score and Z-score. **(B, C)** Chemical structures of cucurbitacin I and temsirolimus. **(E, F)** Representative docking poses and 2D interaction maps for cucurbitacin I bound to CXCL13, IGF1, IL33, and TLR4 (AutoDock/Vina; AlphaFold receptor models; interactions include H-bonds, hydrophobic contacts, and π–π stacking). **(D)** Dot plot summarizing binding energies (kcal/mol) for cucurbitacin I across the four targets (more negative indicates stronger binding). **(H–I**) Analogous docking visualizations for temsirolimus, with **(G)** the corresponding binding-energy summary. All modeled complexes show favorable energies (≤ −5.0 kcal/mol), supporting physical plausibility for target engagement and prioritization for functional testing. DEG, differentially expressed gene.

Utilizing 23 DEGs associated with gut-immune interactions (comprising 9 upregulated and 14 downregulated genes) as input, we employed the “reverse” mode in L1000FWD to independently assess signature reversal for the upregulated and downregulated subsets. Compounds were ranked based on Combined Score and Z-score. The top ten small molecules were identified, with Cucurbitacin I and Temsirolimus consistently achieving the highest rankings and demonstrating the most significant reverse enrichment relative to the query signature (**Figures 12A–C**). After a comprehensive assessment of their pharmacological characteristics and safety data, these two compounds were given priority for subsequent molecular docking investigations and computer simulation evaluations.

### 3.10 Molecular docking analysis of drug–target interactions for IPF treatment

The docking energies (kcal/mol) were determined as follows: for Cucurbitacin I with CXCL13, IGF1, IL33, and TLR4, the values were −6.8, −7.9, −7.7, and −6.7, respectively (**Figures 12D–F**; **Supplementary Table 10**); for Temsirolimus with the same targets, the energies were −7.6, −5.7, −8.5, and −6.4, respectively (**Figures 12G–I**; **Supplementary Table 10**). All complexes demonstrated binding energies of ≤ −5.0, indicating favorable predicted binding conformations in this in silico docking-based assessment. A variety of interaction types, including hydrogen bonds, hydrophobic contacts, and π–π stacking, were observed, supporting the plausibility of potential ligand–protein interactions under the docking model rather than confirming biochemical binding or inhibitory activity. These in silico observations warrant further experimental validation.

### 3.11 Mendelian randomization analysis reveals causal associations between gut microbiome and IPF risk

**Figure 13:**
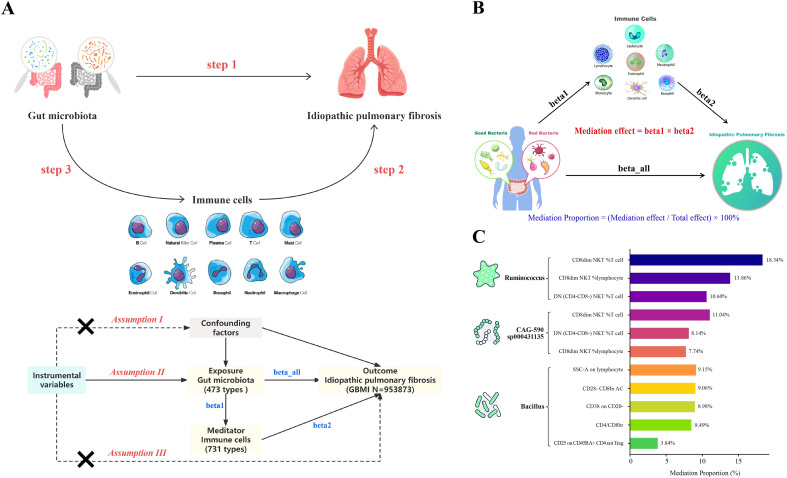
Two-sample Mendelian randomization and immune mediation of gut-microbiome effects on IPF **(A)** Schematic of the three-step MR framework: step 1, causal effects of gut microbiome taxa on IPF; step 2, causal effects of immune cell phenotypes on IPF; step 3, mediation analysis for significant taxa–immune pairs (microbiome → immune → IPF). Instrument selection used genome-wide summary statistics with thresholds P < 1×10^-5^, LD clumping r² < 0.001 (10,000 kb), and F > 10 to exclude weak instruments; primary estimator IVW, with MR-Egger/weighted median and heterogeneity/pleiotropy diagnostics as sensitivity analyses. **(B)** Mediation formulation: total effect (β_all), indirect effect (β_1_×β_2_), direct effect (β_dir = β_all − β_1_×β_2_), and proportion mediated = (β_1_×β_2_/β_all) × 100%. **(C)** Summary of significant mediation proportions for exemplar pairs: Ruminococcus (protective) mediated via NKT-related subsets (e.g., DN [CD4^-^CD8^-^] NKT %T cells; CD8^dim NKT %lymphocytes), CAG-590 sp000431135 via NKT phenotypes, and Bacillus (risk) via multiple immune traits (e.g., CD28^-^CD8 absolute counts, CD4/CD8 ratio, SSC-A on lymphocytes). MR, Mendelian randomization; IVW, inverse-variance weighted; LD, linkage disequilibrium; NKT, natural killer T; SSC-A, side-scatter area.

A two-sample MR analysis revealed directional causality between the gut microbiome and IPF (**Figure 13A**; **Supplementary Table 11**). Specifically, Bacillus was found to have a positive causal effect on the risk of developing IPF (OR = 2.93, 95% CI 1.49–5.79, P = 0.002). In contrast, Ruminococcus and CAG-590 sp000431135 demonstrated negative causal effects (OR = 0.67, 95% CI 0.45–0.99, P = 0.047; OR = 0.78, 95% CI 0.62–0.97, P = 0.029, respectively) (**Supplementary Figure 1**). Within the “gut microbiome (GM) → immune phenotype → IPF” pathway, the protective effect of Ruminococcus was partially mediated by natural killer T (NKT) cell-related subsets, with mediation proportions of 10.60% for DN(CD4-CD8-) NKT %T cells, 13.86% for CD8dim NKT %lymphocytes, and 18.34% for CD8dim NKT %T cells. Conversely, the detrimental effect of Bacillus on IPF was partially mediated through various immune phenotypes, including CD25 on CD45RA+CD4 non-regulatory T cells (3.84%), CD38 on CD20 (8.98%), absolute counts of CD28−CD8 (9.06%), CD4/CD8 (8.49%), and lymphocyte side-scatter area (SSC-A) (9.15%). These findings highlight the critical role of the gut–immune axis in mediating disease risk (**Figures 13B–C**; **Supplementary Table 12**).

### 3.12 *In vitro* and *in vivo* validation of core genes as key players in IPF progression

**Figure 14:**
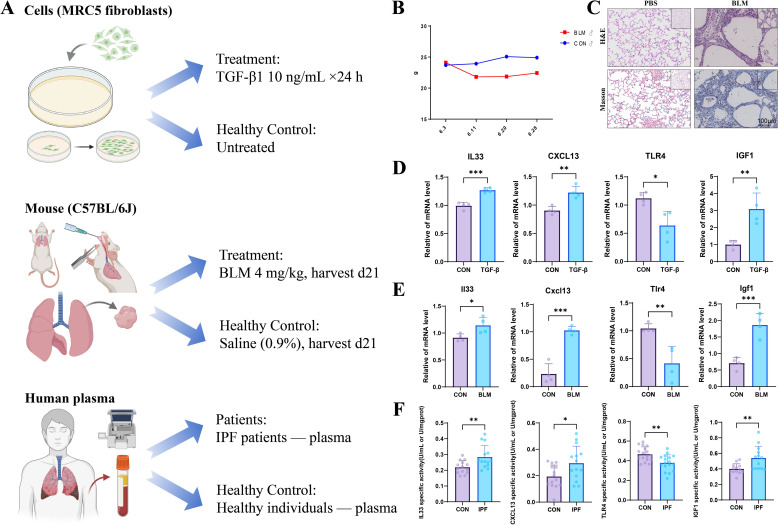
Multidimensional validation of core genes in pulmonary fibrosis models **(A)** Group design for cellular experiments, murine animal models, and human blood samples. **(B)** Changes in body weight of bleomycin-induced IPF and control mice. **(C)** Collagen deposition and lung structural changes in the lungs of fibrotic and control mice. **(D)** Increased expression of IL-33, CXCL13, and IGF-1, and decreased expression of TLR4 in MRC-5 fibroblasts treated with TGF-β1. **(E)** Increased mRNA levels of Il33, Cxcl13, and Igf1, and decreased expression of Tlr4 in bleomycin-treated C57BL/6J mice. **(F)** Elevated plasma levels of IL-33, CXCL13, and IGF-1, and reduced TLR4 levels in IPF patients compared to healthy controls. *P < 0.05, **P < 0.01, and ***P < 0.001.

To investigate the expression dynamics of pivotal target genes associated with pulmonary fibrosis, we devised a comprehensive experimental approach encompassing three distinct dimensions: cellular models, murine lung tissue, and validation using human blood samples (**Figure 14A**). We first evaluated the mRNA levels of core genes in human lung fibroblasts (MRC-5) following TGF-β1 stimulation. After 24 hours of treatment with 10 ng/mL TGF-β1, we observed a significant increase in the expression of IL-33, CXCL13, and IGF-1 compared to untreated controls. In contrast, TLR4 expression was markedly reduced in response to TGF-β1 treatment, suggesting its involvement in regulating fibrosis-related pathways (**Figure 14D**). In the in - vivo model, male C57BL/6J mice were subjected to intratracheal instillation of bleomycin sulfate at a dosage of 4 mg/kg to establish a bleomycin-induced pulmonary fibrosis model. On day 21, lung tissue analysis revealed significant increases in the mRNA expression of il33, cxcl13, and igf1, whereas Tlr4 expression was notably reduced in bleomycin-treated mice compared to controls (**Figure 14E**). Histological examination further confirmed substantial collagen deposition and altered lung architecture in bleomycin-treated mice, as shown by hematoxylin-eosin and Masson’s trichrome staining (**Figures 14B, C**). In clinical validation, plasma samples from IPF patients were analyzed for cytokine levels. Plasma concentrations of IL-33, CXCL13, and IGF-1 were significantly higher in IPF patients compared to healthy controls, while TLR4 levels were lower in the plasma of IPF patients (**Figure 14F**). These findings collectively underscore the dysregulation of IL-33, CXCL13, IGF-1, and TLR4 across cellular, animal, and clinical models, highlighting their potential as biomarkers and their involvement in the progression of IPF.

## 4 Discussion

We conducted an integrative analysis of bulk RNA sequencing, single-cell, and spatial transcriptomic datasets, incorporating machine learning techniques and two-sample MR to systematically elucidate molecular–cellular interactions in IPF within the gut–immune–lung network. This was complemented by experimental validation of core-gene expression. Our findings are consistent with the established model of “epithelial injury—immune disequilibrium—persistent fibroblast activation” and further elucidate upstream signals originating from the gut microbiota, the pulmonary immune network, and the spatiotemporal expression of core genes (45). This establishes a comprehensive framework linking genetic causality to the local microenvironment. Concordant multi-omics analyses identified four core genes—CXCL13, IL-33, TLR4, and IGF-1—as central to immune infiltration and microbiota–metabolic dysregulation, offering novel molecular insights into the progression of fibrosis.

CXCL13 has been identified as a key diagnostic gene. Both single-cell and spatial analyses have demonstrated its significant upregulation at the immune–stromal interface adjacent to fibrotic foci, accompanied by enhanced B cell–related signaling. This pattern is highly consistent with the frequent presence of bronchus-associated tertiary lymphoid structures in lungs affected by IPF (46). The formation and maturation of TLS are dependent on chemokines such as CXCL13, which remodel the local environment to facilitate antigen presentation and B-cell differentiation (47). Numerous histopathological and quantitative studies have detected TLS at various stages of IPF and have linked them to inflammatory activity and the clinical course of the disease (48). By integrating prior literature, it is evident that interactions between CXCL13-expressing tissue-resident T cells and TLS-resident B cells are crucial for maintaining the local humoral immune niche (49). Our data further emphasize the role of the “CXCL13–TLS–B-cell axis” in reshaping the immune microenvironment in IPF.

IL33 expression is elevated in fibroblasts as well as epithelial and endothelial cells, and it is associated with characteristics of mast cells. As an alarmin involved in type 2 inflammation, the IL-33/ST2 signaling pathway enhances mucosal immunity and tissue repair mechanisms (50). In animal models, overexpression of full-length IL-33 or increased ST2 signaling intensifies bleomycin-induced pulmonary fibrosis (51). IL-33 likely contributes to a profibrotic environment indirectly by facilitating cross-lineage communication between mucosal immune cells (such as ILC2s and mast cells) and stromal cells (52). Consistent with this, our communication analyses indicate that IL-33-related signals are predominantly located within immune-stromal interaction pathways rather than isolated intracellular signaling cascades.

TLR4 expression was significantly upregulated in dendritic cells, macrophages, and epithelial cells, and this upregulation was associated with alterations in M2 macrophages and eosinophils. The DAMP–TLR4 axis has the potential to induce a “persistently activated” phenotype in fibroblasts, leading to increased collagen production and the activation of multiple profibrotic pathways (53). Both human and murine studies suggest that the MD2/TLR4 complex plays a crucial role in driving chronic organ fibrosis (54). Nevertheless, specific ligands, such as fragmented hyaluronan, can facilitate the regeneration of epithelial progenitors via TLR4, thereby alleviating severe fibrosis. These cell type- and ligand-dependent effects suggest that a universal inhibition of TLR4 may not be appropriate, and instead, precise targeting of fibroblasts or specific DAMP–TLR4 interactions may be more effective (55). Our findings indicate that TLR4 functions as a signal “amplifier” within immune-stromal interactions, rather than serving as a singular upstream trigger.

IGF1 is upregulated in fibroblasts and specific immune cells, exhibiting a positive correlation with M2 macrophages, activated memory CD4^+^ T cells, and B cells. Previous research has demonstrated that epithelial cells can induce IGF-1 production in alveolar macrophages through TGF-β to facilitate tissue repair (56). However, in fibrotic environments, the IGF-1/IGF1 receptor (IGF1R)–phosphoinositide 3-kinase (PI3K)/protein kinase B (AKT) signaling pathway promotes fibroblast proliferation and ECM production, and is associated with epithelial cell senescence (57). Both *in vitro* and *in vivo* studies suggest that inhibiting IGF1R or its glycosylation control can mitigate fibrosis. Combined with our spatial and single-cell analyses, these findings suggest that IGF-1 functions as a “repair–fibrosis seesaw” regulator. Under conditions of sustained injury and an immune milieu skewed towards M2/SPP1^+^ macrophages and tertiary lymphoid structure activation, IGF-1 is co-opted into a profibrotic program.

The two-sample MR analysis provides genetic evidence supporting the “gut–immune–lung” axis, demonstrating that specific microbial clades have directionally consistent causal effects on the risk of IPF, with significant mediation proportions through NKT-cell or B-cell phenotypes. Existing literature suggests that metabolites such as short-chain fatty acids (SCFAs) mitigate inflammation and fibrosis via G-protein-coupled receptors (GPCRs), epigenetic mechanisms, and barrier functions (58, 59). Recent MR studies also implicate various microbial genera and families in the causation of IPF (60). Moreover, NKT cells are known to facilitate neutrophil recruitment, inflammasome activation, and fibroblast activation in the context of chronic inflammation and fibrosis. Our findings, which highlight the “Ruminococcus–protective–NKT-mediated” and “Bacillus–risky–multi-phenotype immune–mediated” pathways, align with the SCFA production capacity and mucosal immune regulation. However, strain- and host-dependent heterogeneity is probable and necessitates further validation through fecal transplantation and decolonization experiments.

CellChat analysis suggests that the interactions between fibroblasts and innate immune cells are predominantly mediated by the MIF–(CD74+CD44) and CXCL signaling networks. In contrast, macrophages are characterized by active SPP1 and GALECTIN signaling pathways. Independent research identifies SPP1^+^ macrophages as a signature subset in IPF, particularly at fibroblastic foci, where they are linked to collagen deposition (61). The MIF family, through the CD74–CD44 complex, plays a role in B-cell survival and the regulation of multi-organ fibrosis (62). Supported by spatial evidence of epithelial–stromal–immune proximity, we hypothesize that SPP1^+^ macrophages and the MIF axis collectively enhance the “receptor readiness” of fibroblasts. Concurrently, CXCL13 and tertiary lymphoid structures maintain a sustained humoral environment, thereby collectively facilitating the coupling between immune and stromal components (63).

Candidate compounds identified through reverse-signature screening, such as temsirolimus and cucurbitacin-I, were explored only as hypothesis-generating leads rather than confirmed therapeutics, and their relevance is discussed here in terms of pathway-level plausibility rather than target-specific binding or efficacy (64). Specifically, the mTOR/PI3K–AKT and JAK/STAT signaling pathways intersect with IGF-1 signaling and SPP1^+^ macrophages (65, 66), suggesting that pharmacologic modulation of these convergent axes may be conceptually consistent with the immune–stromal programs highlighted by our multi-omics analyses. Furthermore, the interaction between upstream DAMP recognition by TLR4 and downstream NF-κB/SMAD signaling indicates the potential for selective inhibition at the MD2/TLR4 complex or at specific ligand-generation stages (54), although such intervention would require careful context- and cell-type–aware validation given the pleiotropic biology of TLR4 signaling. Importantly, both TLR4 and IL-33 demonstrate cell/ligand-dependent biphasic effects, suggesting that “scenario-specific precision intervention”—such as targeted delivery to SPP1^+^ macrophages or localized drug release within TLS-enriched regions—may be a more rational therapeutic hypothesis than global pathway blockade (67). Numerous studies corroborate the feasibility of these strategies at a conceptual and preclinical level (65, 66, 68). Nevertheless, we emphasize that reverse-signature screening (and any downstream in silico prioritization) provides preliminary, non-confirmatory evidence; therefore, translation will require a rigorous pharmacology and specificity assessment, including promiscuity/PAINS considerations, ADME profiling, and experimental validation in relevant cellular and *in vivo* fibrosis models. However, successful clinical translation will necessitate meticulous design concerning patient stratification, therapeutic windows, and safety considerations.

This study is constrained by batch effects present in public datasets, the predominance of MR instruments derived from European populations, and the incomplete validation of microbiota–host–immune interactions. Future research should incorporate fecal microbiota transplantation, germ-free animal models, spatiotemporal omics perturbations, and human organoid platforms to elucidate causal mechanisms along the gut–immune–fibrosis axis and establish a foundation for individualized precision therapy in IPF.

## 5 Conclusion

This study employs multi-omics data integration and machine learning to elucidate the pivotal roles of gut dysbiosis and immune dysregulation in IPF. It identifies four core genes—CXCL13, IL33, TLR4, and IGF1—as critical for accurate IPF diagnosis, with single-cell and spatial transcriptomics providing insights into their cellular origins and functions. Genetic evidence associates specific gut microbiota with IPF risk through immune pathways, supporting the existence of a “gut–immune–lung” axis in IPF. Modulating the microbiota or restoring immune equilibrium may represent effective therapeutic strategies. Drug screening identified cucurbitacin I and temsirolimus as candidate compounds for further investigation, and molecular docking provided a preliminary in silico, computer-simulation–based assessment of their potential interactions with the core targets. In summary, by integrating multidimensional data, this research clarifies the pathogenesis of IPF and establishes cross-domain connections among the gut ecosystem, immune imbalance, and pulmonary fibrosis. The core molecules and candidate drugs identified herein lay the groundwork for early diagnosis and precision therapy.

## Data Availability

The original contributions presented in the study are included in the article/**Supplementary Material**. Further inquiries can be directed to the corresponding authors.

## Supplementary material

Supplementary Figure 1 Extended two-sample Mendelian randomization results supporting the gut–immune–IPF axis

Supplementary Figure 2 Post-normalization expression distributions for the merged discovery cohorts (GSE32537 and GSE110147) shown by boxplots

Supplementary Figure 3 Principal component analysis (PCA) of the merged discovery cohorts before and after ComBat batch-effect correction

Supplementary Figure 4 Step-by-step workflow for integrated discovery and validation of gut–immune IPF signatures across multi-omics and experimental validation

## Glossary

AUC: Area under the curve
CCL18: C-C motif chemokine ligand 18
CCR: C-C motif chemokine receptor
CypA: Cyclophilin A
CXCL: C-X-C motif chemokine ligand (family)
CXCL13: C-X-C motif chemokine ligand 13
DAMP: Damage-associated molecular pattern
DEG(s): Differentially expressed gene(s)
DN: Double negative
ECM: Extracellular matrix
EDN: Endothelin
ELISA: Enzyme-linked immunosorbent assay
EPC: Edge percolated component
FDR: False discovery rate
GBM: Gradient boosting machine
GBMI: Global Biobank Meta-analysis Initiative
GEO: Gene Expression Omnibus
GO: Gene Ontology
GPCR(s): G protein–coupled receptor(s)
GWAS: Genome-wide association study
IGF-1: Insulin-like growth factor-1
IGF1R: Insulin-like growth factor 1 receptor
IL-17: Interleukin-17
IL-33/IL33: Interleukin-33
ILC2: Group 2 innate lymphoid cell
IPF: Idiopathic pulmonary fibrosis
IV: Instrumental variable
IVW: Inverse-variance weighted
JAK/STAT: Janus kinase/signal transducer and activator of transcription
KEGG: Kyoto Encyclopedia of Genes and Genomes
L1000FWD: L1000 Fireworks Display
LASSO: Least absolute shrinkage and selection operator
LDA: Linear discriminant analysis
LD: Linkage disequilibrium
MAE: Mean absolute error
MAPK: Mitogen-activated protein kinase
MD-2: Myeloid differentiation factor 2
MIF: Macrophage migration inhibitory factor
MR: Mendelian randomization
MR-PRESSO: Mendelian Randomization Pleiotropy RESidual Sum and Outlier
NET: Neutrophil extracellular trap
NF-κB: Nuclear factor kappa-B
NK: Natural killer (cell)
NKT: Natural killer T (cell)
OR: Odds ratio
PCA: Principal component analysis
PI3K: Phosphoinositide 3-kinase
PPI: Protein–protein interaction
QC: Quality control
RNA-seq: RNA sequencing
RT-qPCR: Reverse transcription quantitative polymerase chain reaction
SCFA(s): Short-chain fatty acid(s)
scRNA-seq: Single-cell RNA sequencing
SD: Standard deviation
SE: Standard error
SPP1: Secreted phosphoprotein 1
SSC-A: Side scatter area
ST2: Suppression of tumorigenicity 2 (IL1RL1)
SVM: Support vector machine
t-SNE: t-Distributed Stochastic Neighbor Embedding
TGF-β: Transforming growth factor-β
TLR4: Toll-like receptor 4
TLS: Tertiary lymphoid structure
TNF: Tumor necrosis factor
UMAP: Uniform Manifold Approximation and Projection
WGCNA: Weighted gene co-expression network analysis
XGBoost: Extreme gradient boosting
plsRglm: Partial least squares generalized linear model
