# Supplementary figures and images for: Multi-omics integration and machine learning reveal gut-immune signatures in idiopathic pulmonary fibrosis: insights from bulk RNA-seq, single-cell profiles, spatial transcriptomics, and experimental validation
Source: Front Immunol. 2026 Mar 19;17:1730289. doi: 10.3389/fimmu.2026.1730289 (PMC13043422; doi:10.3389/fimmu.2026.1730289)

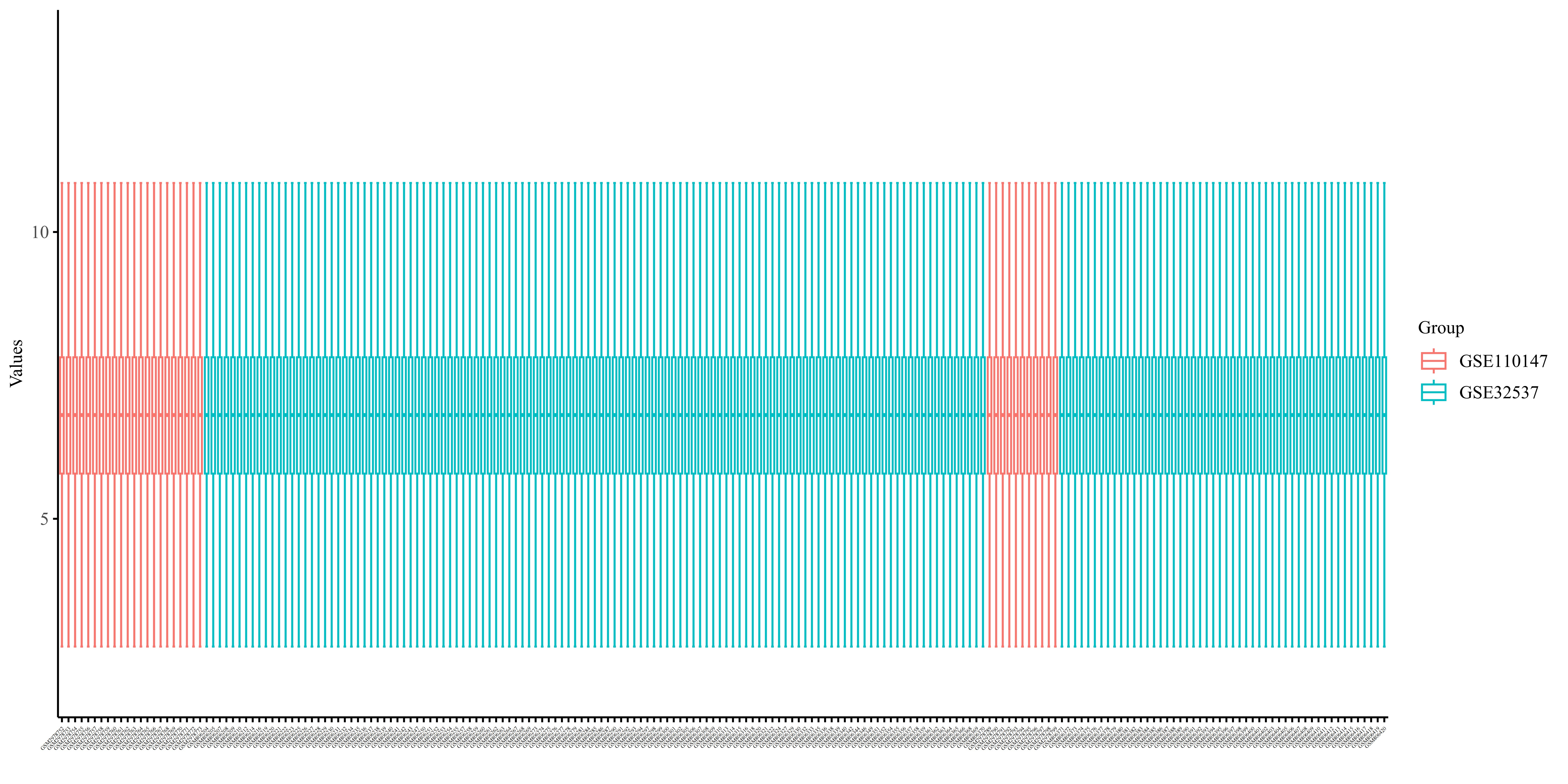

Supplement: Supplementary Figure 2 — Post-normalization expression distributions for the merged discovery cohorts (GSE32537 and GSE110147) shown by boxplots [file Image2.jpeg]

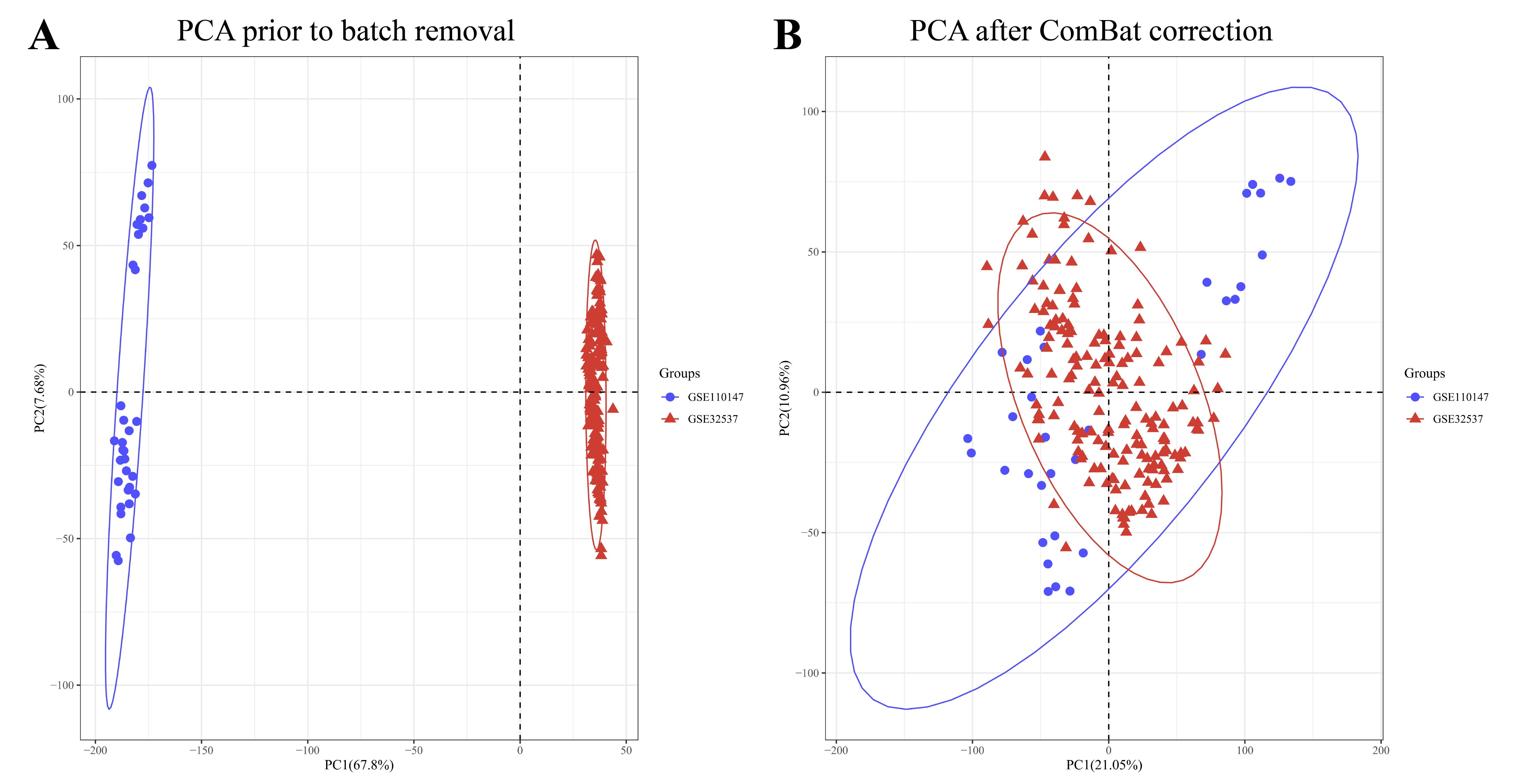

Supplement: Supplementary Figure 3 — Principal component analysis (PCA) of the merged discovery cohorts before and after ComBat batch-effect correction [file Image3.jpeg]

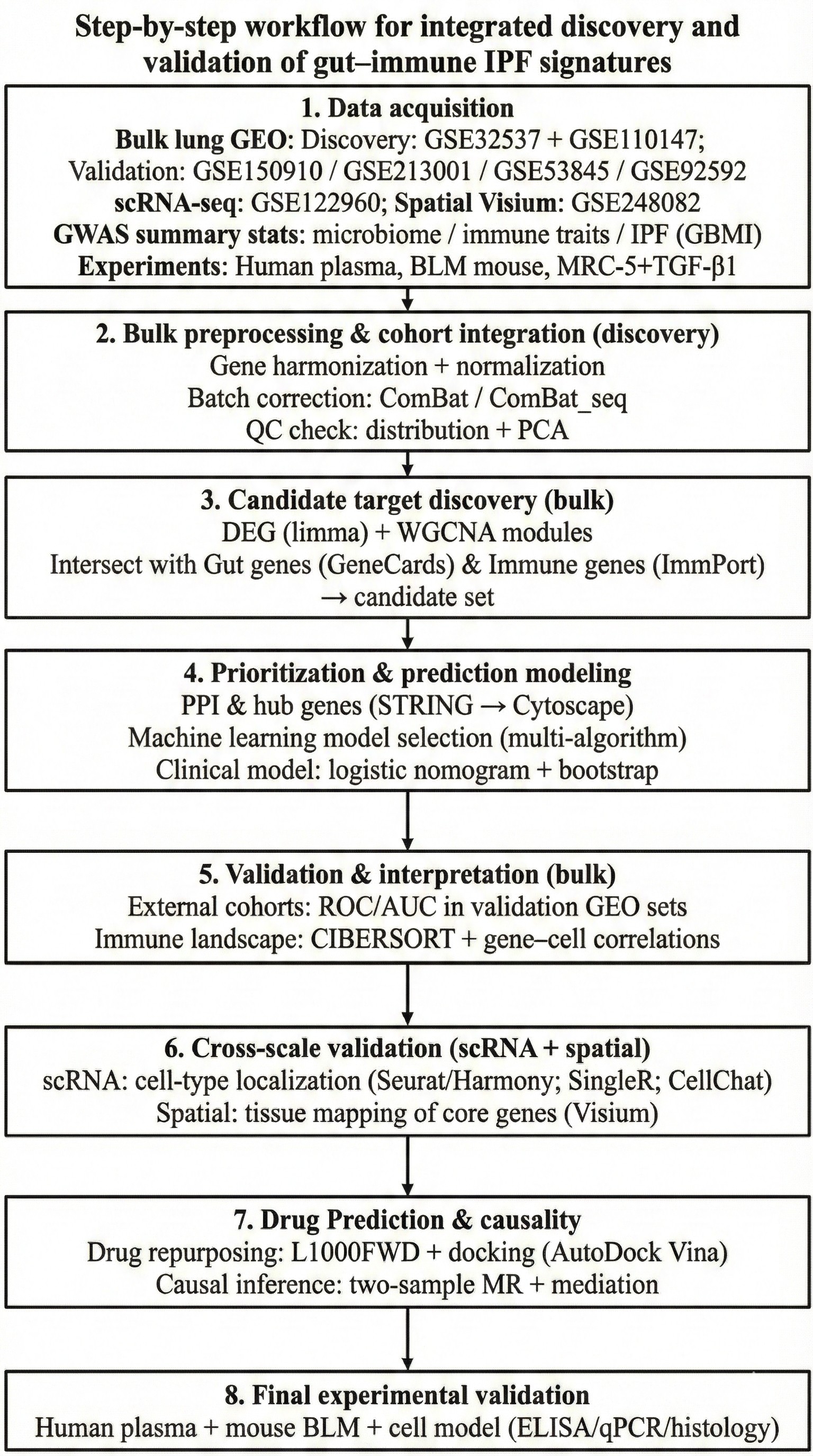

Supplement: Supplementary Figure 4 — Step-by-step workflow for integrated discovery and validation of gut–immune IPF signatures across multi-omics and experimental validation [file Image4.jpeg]
